# Disruption of Chromatin Dynamics by Hypotonic Stress Suppresses HR and Shifts DSB Processing to Error-Prone SSA

**DOI:** 10.3390/ijms222010957

**Published:** 2021-10-11

**Authors:** Lisa Marie Krieger, Emil Mladenov, Aashish Soni, Marilen Demond, Martin Stuschke, George Iliakis

**Affiliations:** 1 Department of Radiation Therapy, Division of Experimental Radiation Biology, University Hospital Essen, University of Duisburg-Essen, 45147 Essen, Germany; lisamarie.krieger@uk-essen.de (L.M.K.); emil.mladenov@uk-essen.de (E.M.); aashish.soni@uk-essen.de (A.S.); martin.stuschke@uk-essen.de (M.S.); 2 Institute of Medical Radiation Biology, University Hospital Essen, University of Duisburg-Essen, 45147 Essen, Germany; marilen.demond@googlemail.com; 3 German Cancer Consortium (DKTK), Partner Site University Hospital Essen, German Cancer Research Center (DKFZ), 45147 Essen, Germany

**Keywords:** DNA double-strand breaks, DNA-damage-response, chromatin, chromatin dynamics, hypotonic stress (HypoS), homologous recombination (HR), nonhomologous end-joining (NHEJ), alternative end-joining (alt-EJ), single-strand annealing (SSA), ionizing radiation (IR)

## Abstract

The processing of DNA double-strand breaks (DSBs) depends on the dynamic characteristics of chromatin. To investigate how abrupt changes in chromatin compaction alter these dynamics and affect DSB processing and repair, we exposed irradiated cells to hypotonic stress (HypoS). Densitometric and chromosome-length analyses show that HypoS transiently decompacts chromatin without inducing histone modifications known from regulated local chromatin decondensation, or changes in Micrococcal Nuclease (MNase) sensitivity. HypoS leaves undisturbed initial stages of DNA-damage-response (DDR), such as radiation-induced ATM activation and H2AX-phosphorylation. However, detection of ATM-pS1981, γ-H2AX and 53BP1 foci is reduced in a protein, cell cycle phase and cell line dependent manner; likely secondary to chromatin decompaction that disrupts the focal organization of DDR proteins. While HypoS only exerts small effects on classical nonhomologous end-joining (c-NHEJ) and alternative end-joining (alt-EJ), it markedly suppresses homologous recombination (HR) without affecting DNA end-resection at DSBs, and clearly enhances single-strand annealing (SSA). These shifts in pathway engagement are accompanied by decreases in HR-dependent chromatid-break repair in the G_2_-phase, and by increases in alt-EJ and SSA-dependent chromosomal translocations. Consequently, HypoS sensitizes cells to ionizing radiation (IR)-induced killing. We conclude that HypoS-induced global chromatin decompaction compromises regulated chromatin dynamics and genomic stability by suppressing DSB-processing by HR, and allowing error-prone processing by alt-EJ and SSA.

## 1. Introduction

Each cell has to deal with tens of thousands of DNA lesions every day [1] that can cause mutations endangering genomic stability. One particularly consequential form of DNA damage is the DNA double-strand break (DSB). Although DSBs form infrequently, even after exposure to ionizing radiation (IR), they pose high risk for the cell [2,3], as error-prone processing leads to cell death or carcinogenesis. Cells have evolved different repair pathways to process DSBs, all with the ability to structurally restore the DNA molecule, but each operating with different fidelity and bearing different risks to the genome [4].

Homologous recombination (HR) uses the sister chromatid as a template, and is the only DSB repair pathway that can restore the original sequence at the DSB site. End-joining pathways (classical nonhomologous end-joining (c-NHEJ) and alternative end-joining (alt-EJ)), as well as single-strand annealing (SSA), are associated with sequence alterations, insertions or deletions at the junction, and can cause the formation of chromosomal translocations [4]. Cells have also evolved highly sophisticated and complex signaling mechanisms to detect DSBs and promote their repair, collectively known as the DNA-damage-response (DDR) [2,5]. When components of this signaling and repair network are defective, hypersensitivity to DNA damaging agents and diverse disease phenotypes develop [2].

In cells, DSBs are induced in the context of chromatin, and it is long recognized that the characteristics and dynamics of chromatin in the vicinity at the time of induction modulate the emanating DDR and regulate DSB processing. There is evidence that open chromatin facilitates DDR and DSB repair, while hypercondensed chromatin suppresses DDR and alters the balance among active DSB repair pathways [6]. Given the plurality and mechanistic diversity of pathways processing DSBs, it is important to understand how open versus closed chromatin favors or disfavors the function of each one of them [7]. 

Model systems using endonuclease-induced DSBs reveal the prevalence for HR in transcriptionally active genes that typically reside in open chromatin, while c-NHEJ preferentially engages in noncoding sequences frequently found in condensed chromatin [8,9,10]. Moreover, chromatin changes its dynamics upon induction of DSBs and adapts its pre-existing state to optimize processing. Chromatin remodeling in the form of relaxation is thought to be beneficial, as it facilitates access of repair proteins [11,12,13]. Thus, mouse heterochromatic regions in G_2_-phase cells and drosophila pericentromeric heterochromatin can still engage HR by relocating DSBs to euchromatic regions [10,14,15]. Heavy-ion-induced DSBs also relocate to the periphery of heterochromatic centers, again to engage HR [14,16,17]. Notably, disruption of this relocation shifts repair to error-prone pathways such as SSA [14]. 

Chromatin relaxation after DSB induction and its subsequent recondensation following DNA repair are mediated, among other mechanisms, by chromatin remodelers classified into four subfamilies: the imitation switch (ISWI), the chromodomain DNA helicase-binding (CHD), the switch/sucrose nonfermentable (SWI/SNF) and the INO80 subfamilies [18]. Histone acetylation also plays an important role in chromatin relaxation, and indeed acetyltransferase TIP60, a subunit of NuA4 HAT complex β, locally relaxes chromatin upon DSB induction [19]. Another subunit of NuA4, the motor ATPase p400, helps to further increase the accessibility of DNA to repair proteins by destabilizing histone-DNA interactions [20]. 

Despite evidence for local chromatin relaxation upon DSB induction to facilitate repair, other studies report chromatin condensation [12], and variants of the heterochromatin protein 1 (HP1) accumulate at DSB sites [21]. Furthermore, gene silencing mediated by hypermethylation at CpG islands [22], as well as suppression of RNA polymerase II activity and abrogation of transcription-coupled chromatin decondensation [23], suggest chromatin condensation in the vicinity of DSBs. Condensation of chromatin during DSB repair may be beneficial by providing positional stability within the nucleus, as loss of local position-restrains increases genomic instability and leads to translocation formation [12,24]. There is also evidence that the local effects of DSBs on chromatin are cell cycle dependent and reduced in noncycling cells [25,26]. Finally, decondensed chromatin is more sensitive to breakage by IR than condensed chromatin [27].

Collectively, the above outline suggests that the pre-existing chromatin environment influences DSB induction and repair pathway engagement, and that its subsequent regulated modulation profoundly contributes to DSB processing. However, available information is insufficient for generating a consistent model either for the pre-induction or the post-induction stage, which suggests that important determinants and mechanisms await elucidation. 

The present work attempts to partly fill this gap by analyzing how post irradiation exposure of cells to hypotonic stress (HypoS) by incubation in low salt, hypotonic growth medium modulates DDR and DSB-processing. HypoS and treatments in anisotonic media in general generate a plethora of effects, including global decondensation of chromatin, and allow analysis of these effects on radiation responses [28,29,30,31,32]. Physiologically, decreases in inorganic ions in the extracellular fluid are known to generate regulatory volume decreases [33], and reductions in the intracellular ionic strength alter the structure and function of biological macromolecules [34,35]. There are several pathophysiological conditions causing changes in cell volume, such as hypoxia/ischemia, hyponatremia, hypothermia and increases in extracellular K^+^ concentration [33]. 

An anisotonic environment is also known to profoundly radiosensitize cells to IR [28,29,30,31], to impair DSB repair efficiency [29] and to interrupt repair of potentially lethal damage (PLD) [29,36]. Aneuploidy, a characteristic of many cancer cells, also causes stress responses comparable to HypoS [37]. Notably, the mechanisms underpinning these impressive responses remain uncharacterized, despite recent advances in our understanding of DDR. Here, we study the effect of HypoS on radiation response and report striking effects on DSB repair pathway engagement and genomic stability that help advance our understanding of how HypoS affects DDR by disrupting chromatin dynamics.

## 2. Results

### 2.1. HypoS Causes Chromatin Decondensation

**Figure 1:**
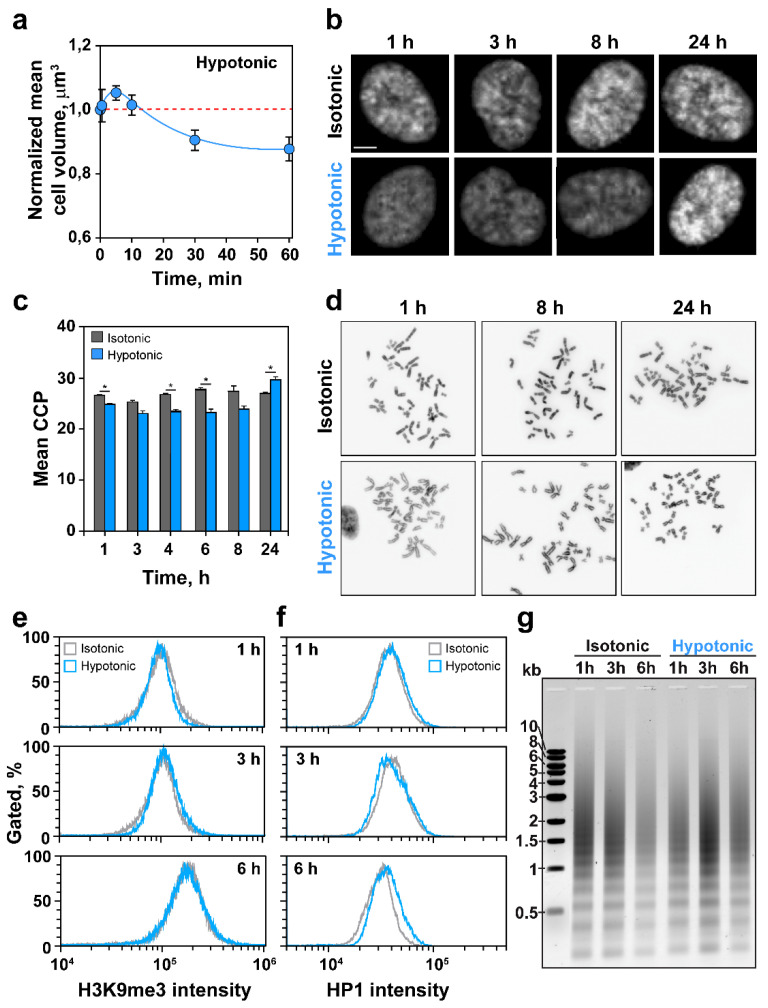
HypoS decondenses chromatin. Exponentially growing RPE cells were incubated in isotonic or hypotonic medium as indicated. (**a**) Mean cell volume of cells exposed to HypoS as a function of time, measured in a cell counter/volume analyzer. Data is normalized to the cell volume measured at 0 h. Mean ± standard error (SE) from four independent experiments is plotted. (**b**) Representative images of DAPI stained nuclei showing chromatin decondensation and recondensation after HypoS (lower panel). Scale bar: 5 µm. (**c**) Mean CCP values as a function of time after HypoS (blue bars) and isotonically maintained controls (grey bars). Mean ± SE calculated from two independent experiments. The significance of differences is indicated by * *p* < 0.05. (**d**) Representative images of metaphase chromosomes in cells exposed to HypoS or maintained in isotonic medium for the indicated times. (**e**,**f**) Flow cytometry (FC) analysis of H3K9me3 (**e**) and HP1 (**f**) in RPE cells exposed to HypoS or maintained in isotonic medium for the indicated times. (**g**) Micrococcal Nuclease (MNase) digestion patterns of RPE cell DNA exposed to HypoS or maintained in isotonic medium for the indicated times.

In the present work, we use immortalized human retinal epithelial cells (RPE) as a model cell system representing normal cells. Key findings are confirmed using immortalized human fibroblasts, as well as tumor cell lines of epithelial or mesenchymal origin. This approach serves to underpin the generality of our observations in normal and tumor cells. Transfer of actively growing RPE cells to hypotonic medium stresses cells by causing water influx that increases their volume by about 5% within 5 min (Figure 1a). This increase is followed by a regulatory volume decrease within ~10 min that normalizes and even overcompensates cell volume by ~10% at 1 h. Since we are interested in the effects of HypoS on DDR and DSB repair, we investigated effects on chromatin using image analysis of DAPI stained nuclei. Figure 1b shows that nuclei of cells maintained in isotonic medium display numerous brightly stained areas of high DNA content reflecting condensed chromatin, including heterochromatin. Notably, high DAPI-intensity areas are reduced after 1 h of incubation in hypotonic medium and are barely discernible after 3–8 h, suggesting chromatin decondensation. They re-emerge and show overcompensation 24 h later. 

To quantify the chromatin decondensation shown in Figure 1b, we employed image analysis as outlined under 4.6. The method applies the Sobel edge-detection algorithm to images of DAPI-stained nuclei. The specific code developed by Irianto et al. [38] calculates the edge density of each nucleus to estimate the mean chromatin condensation parameter (CCP) (see Figure S1a for an example of results at different steps in this analysis). Figure 1c shows mean CCP values of nuclei incubated in isotonic and hypotonic medium for the indicated times. The results confirm the decondensation of chromatin that is visually evident in Figure 1b. 

We also analyzed HypoS consequences on mitotic chromosomes that reflect the highest condensation state of chromatin throughout the cell cycle. Figure 1d shows metaphases prepared after different times of incubation in hypotonic medium. In direct analogy to the response of interphase chromatin, chromosomes appear longer than in controls at 1 and 8 h, and shorter at 24 h. Chromosome length quantification in this image confirms these trends (Figure S1b,c). 

Thus, HypoS affects cell volume and chromatin condensation state with markedly different kinetics. Whereas changes in cell volume occur within minutes, and compensatory responses are evident at 30 min, chromatin decondensation is present after about 1 h and is maintained for ~8 h, with over-compensation evident at 24 h.

The global decondensation of chromatin detected in the above experiments may be a passive response to HypoS or a regulated response similar to that accompanying the activation of transcription and other cellular processes – or a combination of both processes. Since Figure 1b suggests a reduction in heterochromatic regions after HypoS, we measured the levels of heterochromatin markers in cells transferred to hypotonic medium: H3K9me3 and HP1. The flow cytometry (FC) results in Figure 1e show that HypoS fails to reduce H3K9me3 in up to 6 h of incubation. The results in Figure 1f also show that HP1 levels remain practically unchanged in cells exposed to HypoS.

Chromatin relaxation associated with gene activation is frequently accompanied by changes in the positioning of nucleosomes, detectable by changes in the patterns of DNA digestion by Micrococcal Nuclease (MNase) treatment. We therefore compared global MNase sensitivity between cells incubated in hypotonic or isotonic media. Figure 1g shows no detectable changes in chromatin digestion patterns within each sample at different times after HypoS. Intensity differences between the lanes reflect fluctuations in DNA recovery from the treated samples, are random, and are not consistent with HypoS effects. We conclude that the low ionic environment generated by HypoS passively decondenses chromatin without detectably utilizing mechanistic inputs known from regulated chromatin decondensation responses.

### 2.2. Effects of HypoS on Cell Proliferation and Cell Cycle Progression

**Figure 2:**
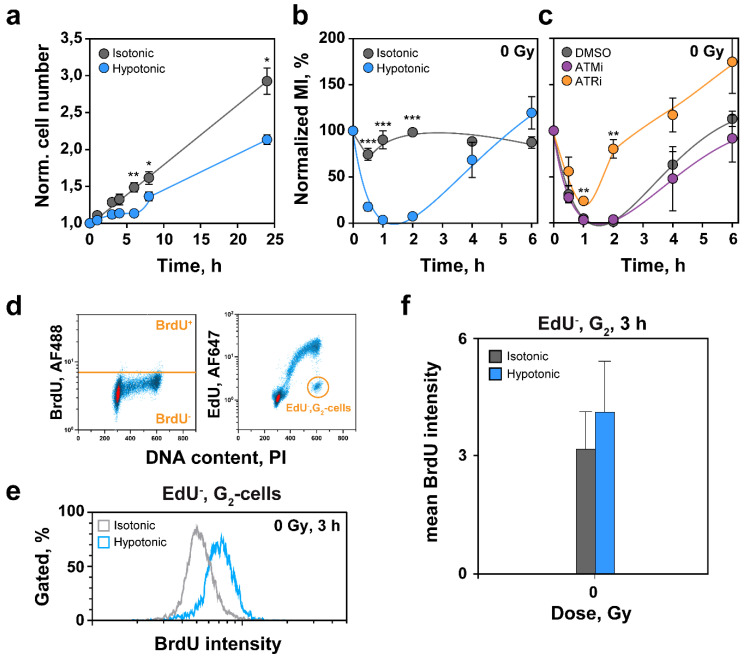
HypoS transiently inhibits cell proliferation. (**a**) Cell proliferation of RPE cells transferred to isotonic (grey symbols) or hypotonic medium (blue symbols). Values are normalized to those measured at 0 h. (**b**) Normalized percent-MI as a function of time for RPE cells exposed to HypoS or maintained in isotonic medium. (**c**) As in (**b**), for cells exposed to HypoS, pretreated for 1 h and then maintained for the duration of experiment with specific inhibitors of ATM (ATMi: KU-55933), ATR (ATRi: VE-821) or solvent (DMSO). Plotted is the mean ± SE from three to five independent experiments. Error bars are visible when larger than the symbols used. (**d**) Outline of the three-parametric FC analysis used to determine ssDNA in G_2_-phase cells. In the left panel, BrdU signal is plotted against the PI signal, and in the right panel EdU against the PI signal. Gates define the populations selected for ssDNA analysis (EdU negative (EdU^−^), G_2_-phase). (**e**) BrdU intensity plots measured by FC in RPE cells exposed to HypoS or maintained in isotonic medium for 3 h. Plots represent EdU^−^, G_2_-phase cells selected using the gating shown in (**d**). (**f**) Arithmetic means of AF488 signal intensity for BrdU measured by FC and gated as in (**d**) of RPE cells maintained in isotonic (grey bar) and hypotonic medium (blue bar) for 3 h. Means and errors are calculated from three independent experiments. Differences reaching statistical significance between isotonic and hypotonic treated cells are marked with * *p* < 0.05, ** *p* < 0.01 and *** *p* < 0.001.

We investigated HypoS effects on cell proliferation and transition through specific phases of the cell cycle. In line with the transient nature of effects on cell volume and chromatin decondensation, HypoS caused a transient inhibition of cell proliferation during the first 6 h (Figure 2a). Indeed, cell proliferation was similar in hypotonically treated and control cells between 8 and 24 h, despite continuing adjustments in chromatin condensation state during this period (see Figure 1b–d). 

To determine the nature of HypoS-induced transient growth inhibition, we analyzed cell progression to mitosis by measuring mitotic index (MI) via H3pS10 analysis by FC. Notably, the MI dropped abruptly during the first hour after HypoS and recovers at 6 h (Figure 2b). Since this response resembles the G_2_-checkpoint activated after exposure of cells to IR, and because the latter is regulated by ATM and ATR [39,40], we inquired whether similar mechanisms underpin HypoS responses. Figure 2c shows that inhibition of ATM with KU-55933 (ATMi), did not alter the response to HypoS. This is surprising but very interesting because HypoS is known to activate ATM [41].

Notably, inhibition of ATR with VE-821 (ATRi), reduced the magnitude of the initial MI-drop and accelerated its recovery, suggesting that HypoS-induced ATR activation partly suppresses cell progression to mitosis. Since canonical ATR activation requires the generation of ssDNA, we quantified ssDNA levels via BrdU signal intensity, specifically in G_2_-phase cells, using the three-parameter FC methodology described earlier [39,40]. Figure 2d shows the gates employed in the analysis and Figure 2e representative BrdU signal intensity distributions in cells maintained in hypotonic or isotonic medium. The results in Figure 2f show an increase in BrdU signal in cells exposed to HypoS, which hint at increased ssDNA levels and ATR activation. We suppose that HypoS suppresses mitotic entry partly by activating ATR through the formation of ssDNA. The mechanism underpinning this increase and its possible connection to DNA replication requires further experimentation.

### 2.3. Effects of HypoS on DDR

**Figure 3:**
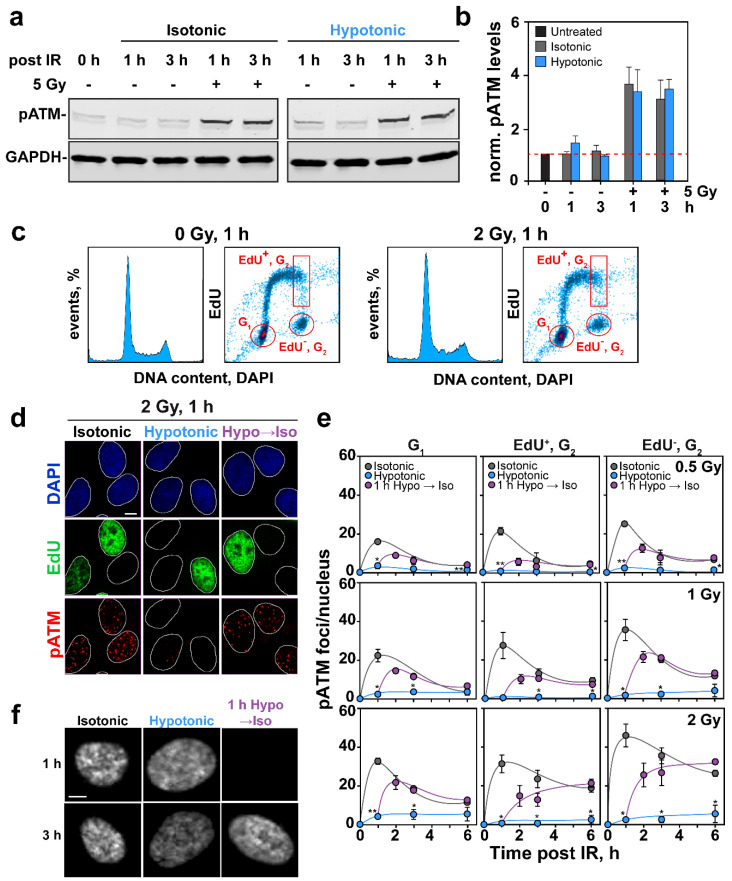
Normal ATM activation but suppressed pATM foci formation after IR in HypoS exposed cells. (**a**) Western blot analysis of pATM in RPE cell extracts as a function of time after irradiation (5 Gy) and incubation in isotonic or hypotonic medium as indicated. GAPDH served as loading control. (**b**) Densitometric analysis of pATM band intensity in a. Plotted is protein level normalized to loading control. Mean ± SE is calculated from three independent experiments. (**c**) Representative histograms and dot plots depicting the gating employed to analyze pATM foci by quantitative image-based cytometry (QIBC) in EdU^−^, G_2_-phase cells. (**d**) Representative images of DAPI-stained RPE nuclei showing EdU incorporation and pATM foci formation at 1 h after 2 Gy in cells exposed to HypoS or maintained in isotonic medium. Results of cells maintained for 1 h in hypotonic medium and analyzed after transferring to isotonic medium are also shown. Scale bar: 5 µm. (**e**) Values of pATM foci formation after background (nonirradiated) subtraction as a function of time, analyzed by QIBC, in G_1_-phase cells, EdU positive (EdU^+^), G_2_-phase cells and EdU^−^, G_2_-phase cells maintained after IR in isotonic (grey symbols) or hypotonic medium (blue symbols). Violet symbols show results of cells treated hypotonically post IR for 1 h and then transferred to isotonic medium. Data represent the mean ± SE from two experiments. Error bars are visible only when larger than the symbols. (**f**) Representative images of DAPI stained RPE nuclei showing chromatin decondensation after HypoS (middle) and recondensation after transfer to isotonic medium 1 h later (right). Scale bar: 5 µm. Differences reaching statistical significance between isotonically and hypotonically treated cells are marked with * *p* < 0.05 and ** *p* < 0.01.

We inquired how HypoS affects the development of DDR in irradiated cells. To exclude HypoS effects on the induction of DSBs, we transferred cells to hypotonic medium immediately after IR. Activation of ATM is an early step in DDR that is conveniently quantified by following its autophosphorylation at Serine-1981 to generate ATM-pS1981 (pATM). It was previously reported that HypoS, and other treatments modifying chromatin, activate ATM by this measure in the absence of IR [41]. We studied ATM activation by Western blotting in RPE cells exposed to 5 Gy and maintained under isotonic or hypotonic conditions. Figure 3a confirms a weak ATM activation in nonirradiated cells exposed to HypoS, which is confirmed by the densitometric analysis of three similar gels in Figure 3b. Notably, exposure to IR induced a robust activation of ATM that persisted for up to 3 h. This response remained unchanged in cells incubated in hypotonic medium suggesting efficient ATM activation after HypoS.

Analysis of pATM foci formation and decay in irradiated cells exposed to HypoS using quantitative image-based cytometry (QIBC) as described earlier [39,40] gave a surprising result (see Figure 3c for gating details). While pATM foci developed quickly in cells maintained in isotonic medium (Figure 3d,e), foci development appeared strongly suppressed in cells incubated in hypotonic medium at all doses and phases of the cell cycle investigated. Figure 3d shows representative nuclei processed as required for QIBC analysis [39,40] and treated as indicated. Figure 3e shows complete kinetics of pATM foci development and decay in RPE cells exposed to 0.5, 1 and 2 Gy and analyzed in G_1_ and G_2_-phases of the cell cycle, while discriminating in G_2_-phase between cells irradiated in S-phase (EdU^+^) and cells irradiated in G_2_-phase (EdU^−^). It is evident that the apparent suppression of pATM foci development in cells exposed to HypoS persisted for the 6 h of observation. Notably, the suppression of pATM foci after HypoS was readily reversible, and foci promptly reappeared after transfer of cells to isotonic medium (Figure 3d,e).

Since analysis by Western blot (Figure 3a) showed normal activation of ATM in irradiated cells exposed to HypoS, we considered the possibility that the absence of visible pATM foci in Figure 3d,e is secondary to HypoS-induced chromatin decondensation, i.e., ATM is present at DSBs at normal levels but becomes dispersed on decondensed chromatin, failing to appear as discernible foci. If this hypothesis holds, chromatin should promptly recondense upon transfer of hypotonically treated cells to isotonic medium to allow the prompt reappearance of pATM foci observed in Figure 3d,e. Indeed, the CCP analysis in Figure 3f shows widespread chromatin rearrangement 2 h after returning HypoS exposed cells to isotonic medium. We therefore suggest that, following HypoS exposure, chromatin decondensation suppresses the organization of pATM in a visible focus.

**Figure 4:**
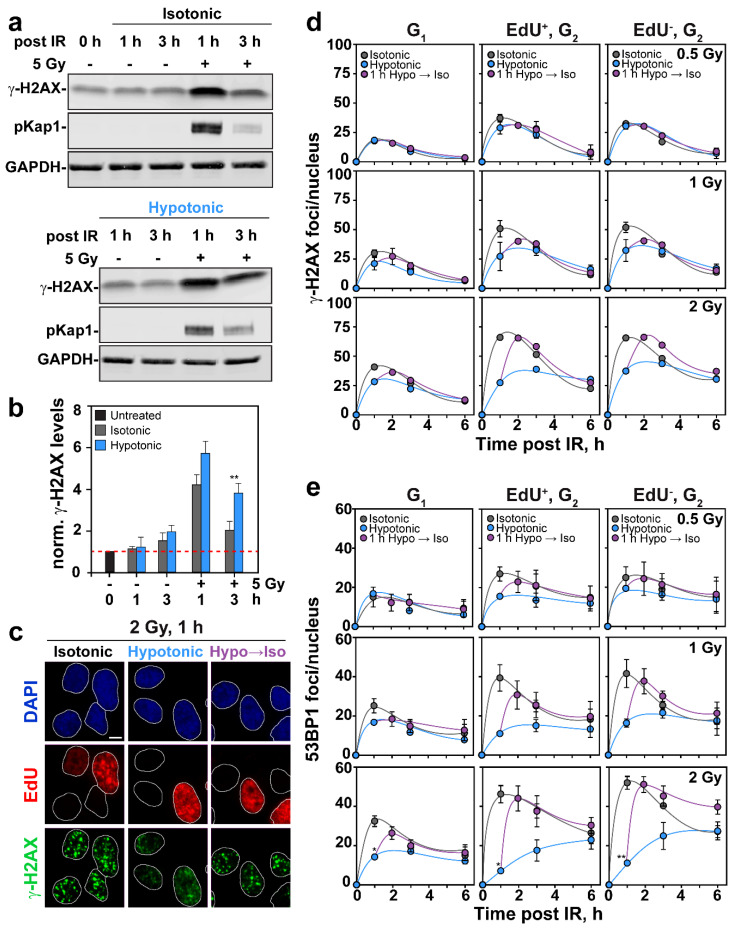
HypoS suppresses formation of γ-H2AX and 53BP1 foci in an IR-dose and cell cycle phase-dependent manner. (**a**) Western blot analysis of γ-H2AX and pKap1 in extracts of RPE cells as a function of time after exposure to HypoS and 5 Gy. GAPDH served as loading control. (**b**) Densitometric analysis of γ-H2AX band intensity in a. Plotted is protein level normalized to loading control. Means ± SE from two independent experiments are shown. (**c**) Representative images of DAPI-stained RPE nuclei showing EdU incorporation and γ-H2AX foci formation after 1 h of HypoS and 2 Gy. Images of cells transiently (1 h) exposed to hypotonic medium are also shown. Scale bar: 5 µm. (**d**) QIBC analysis of γ-H2AX foci formation after background (nonirradiated) subtraction in G_1_-phase cells, EdU^+^, G_2_-phase cells and EdU^−^, G_2_-phase cells maintained in isotonic (grey symbols) or hypotonic medium (blue symbols) after exposure to the indicated IR doses. Violet symbols show kinetics of foci formation in cells that were treated hypotonically post IR for 1 h and then transferred to isotonic medium. Data represents means ± SE from two experiments (except for 2 Gy where *n* = 1). Error bars are visible when larger than the symbols used. (**e**) As in (**d**) for 53BP1. Differences reaching statistical significance between isotonic and hypotonic treated cells are marked with * *p* < 0.05 and ** *p* < 0.01.

A key target of ATM is the histone variant H2AX, whose phosphorylation on nucleosomes in the vicinity of DSBs generates γ-H2AX. Analysis of global γ-H2AX levels by Western blotting in cells exposed to 5 Gy showed the expected prompt increase at 1 h and a decay at 3 h (Figure 4a). HypoS enhanced slightly γ-H2AX formation at 1 h and suppressed its decay at 3 h. Figure 4b shows the densitometric analysis of similar gels from two experiments that confirms these conclusions. Thus, in cells exposed to HypoS, ATM activation occurred at nearly normal levels and led to almost normal levels of γ-H2AX formation. In line with nearly normal levels of ATM activation following HypoS, phosphorylation of Kap1 protein (pKap1), another well-known target of ATM, occurred at virtually normal levels (Figure 4a).

Analysis of γ-H2AX foci formation following HypoS using cell cycle specific QIBC in cells exposed to 0.5, 1 and 2 Gy showed slightly diverging effects compared to Western blotting. Indeed, after exposure to 0.5 Gy, HypoS had no detectable effect on γ-H2AX foci formation and decay in cells irradiated and analyzed in G_1_, or in cells analyzed in G_2_-, but irradiated either in S-phase (EdU^+^) or G_2_-phase (EdU^−^). However, in cells exposed to 1 Gy, and particularly in cells exposed to 2 Gy, HypoS suppressed γ-H2AX foci formation, more pronouncedly in cells irradiated in S or G_2_-phase. When γ-H2AX foci formation was suppressed by HypoS, it could be reversed by transferring cells to isotonic medium (Figure 4d). We postulate that the suppression of γ-H2AX foci formation after HypoS at doses above 2 Gy reflects the above discussed chromatin decompaction. The higher susceptibility shown above of pATM than γ-H2AX foci to HypoS after IR, may reflect the different forms of integration of the two proteins to chromatin.

The phosphorylation-mediated signaling cascade of DDR on chromatin is followed by a ubiquitin signaling cascade that culminates with the recruitment to DSBs of the mediator protein 53BP1 [42]. We analyzed, therefore, the effect of HypoS on 53BP1 foci formation in irradiated RPE cells using the cell cycle-specific method described above for pATM and γ-H2AX. Figure 4e shows that IR-induced 53BP1 foci formation responded to HypoS similarly to γ-H2AX although the effect was slightly larger overall: It became stronger with increasing IR dose and was more pronounced in cells irradiated in S- and G_2_-phase of the cell cycle. Here again, the suppression in 53BP1 foci formation by HypoS was reversible. As for pATM and γ-H2AX foci formation, we postulate nearly normal recruitment of 53BP1 to chromatin that cannot be directly visualized as a consequence of global decondensation of chromatin.

To rule out cell line specificity in the above observed effects of HypoS treatment, we analyzed similar endpoints using QIBC in U2OS, A549 and 82-6 hTert cells. Figure S2a–c show that while the overall HypoS response to foci formation was rather general, its magnitude fluctuated among cell lines. Thus, U2OS cells showed a response similar to RPE cells for pATM and γ-H2AX foci formation, but a stronger response for 53BP1. A549 cells showed a milder response than RPE cells for pATM and 53BP1, but a stronger response for γ-H2AX. Finally, the human fibroblast cell line 82-6 hTert showed a mild effect on pATM and γ-H2AX foci formation, but a complete suppression of 53BP1 foci formation. Considering that the HypoS effects on DDR foci development actually derive from the associated decondensation of chromatin rather than an actual suppression of DDR, the differences among cell lines may reflect differences in chromatin organization and dynamics.

### 2.4. Effects of HypoS on c-NHEJ and alt-EJ

We next inquired how HypoS affects the processing of DSBs and particularly the engagement of available DSB repair pathways, HR, c-NHEJ, alt-EJ and SSA. Although the γ-H2AX signal and foci development and decay kinetics presented above suggest such effects, the described scoring limitations and the inability to carry out repair pathway-specific analysis, led us to opt for alternative approaches.

The first experiments employed pulsed-field gel electrophoresis (PFGE). This DSB detection method has the distinct advantage that it measures the physical presence of DSBs in the genome, rather than the DDR response they elicit [43], but is characterized by low sensitivity that requires high IR doses. We have recently shown that the highly efficient DSB processing observed in unperturbed wild type cells by PFGE at high IR doses largely reflects the function of c-NHEJ, as resection dependent pathways, and particularly HR, are strongly suppressed [44].

**Figure 5:**
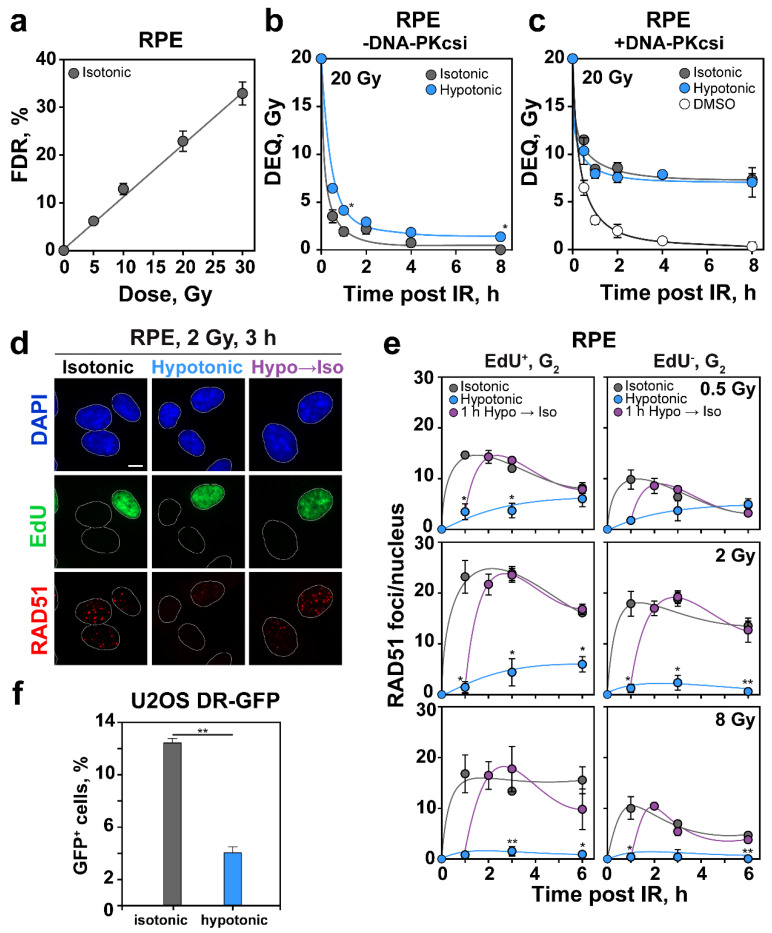
HypoS strongly suppresses HR and causes modest inhibition on c-NHEJ and alt-EJ. (**a**) Dose-response for DSB induction measured by PFGE in RPE cells. The fraction of DNA released (FDR) from the well into the lane is plotted against the IR dose applied. (**b**) Cells were exposed to 20 Gy prior to incubation in isotonic or hypotonic medium. The equivalent dose (DEQ) was calculated using the corresponding dose response curve from the FDR measured at each time point. (**c**) As in (**b**) for cells treated with DNA-PKcsi (5 µM NU7441) 1 h before IR. Data in (**b**,**c**) represent means and SE from eight determinations in two experiments. Error bars are visible when larger than the symbols used. (**d**) Representative images of DAPI-stained nuclei showing EdU incorporation and RAD51 foci formation at 3 h post 2 Gy in isotonically, hypotonically and transiently hypotonically (1 h) treated cells. Scale bar: 5 µm. (**e**) Kinetics of RAD51 foci formation after background (nonirradiated) subtraction analyzed by QIBC in EdU^+^ and EdU^−^ G_2_-phase cells maintained in isotonic or hypotonic medium after IR. Violet symbols show results of cells treated hypotonically for 1 h post IR before transfer to isotonic medium. (**f**) GFP positive (GFP^+^) cells in the U2OS DR-GFP reporter cell line, 24 h after transfection with an I-*Sce*I expressing plasmid followed by incubation in hypotonic (blue bar) or isotonic (grey bar) medium. Means ± SE from two independent experiments are shown. In all plots the significance of differences between isotonic and hypotonic conditions is indicated by the asterisk: * *p* < 0.05 and ** *p* < 0.01.

Figure 5a shows the dose response for DSB induction, drawn as fraction of DNA released (FDR) as a function of IR dose, and estimated by analyzing the corresponding gels as outlined in Figure S3a (gel on the left). This form of dose response curve was generated in every experiment and used in repair experiments to estimate remaining DSBs as a function of time (Figure S3a; gel on the right) as dose equivalent, DEQ, calculated as outlined under 4.5. Figure 5b shows the expected fast DSB processing by c-NHEJ as rapid reduction in DEQ with progressing repair time. Incubation of cells after IR in hypotonic medium slightly delayed DSB processing (see Figure S3b for analysis of the kinetics under different incubation conditions).

Since our earlier work detected promotion of alt-EJ in cells incubated in hypotonic medium [45], we treated RPE cells with NU7441 to inhibit DNA-PKcs, suppress c-NHEJ and bring alt-EJ to the fore. The results in Figure 5c show that alt-EJ was only marginally affected under hypotonic conditions in these cells, although mathematical analysis detected half time changes that failed to reach statistical significance (Figure S3b).

### 2.5. HypoS Profoundly Suppresses HR

To evaluate rather specifically the effects of HypoS on HR, we quantified formation and decay of RAD51 foci using cell cycle specific QIBC [39,40], and analyzed separately the effects of HypoS on HR in cells irradiated in G_2_-phase (EdU^−^) versus cells irradiated in S-phase (EdU^+^). Since we recently reported [44] a strong dependence of HR engagement on IR-dose, with nearly a 50% contribution below 0.5 Gy, but undetectable contribution above 10 Gy, we also analyzed different IR doses. Figure 5d shows examples of nuclei stained for this analysis, whereas Figure 5e shows the kinetics of RAD51 foci development and decay in G_2_-phase cells for EdU^−^ and EdU^+^ cells exposed to 0.5, 1 and 2 Gy. In control cells (Figure 5d,e) RAD51 foci developed within 1–3 h after IR and decayed at later times in an IR-dose-dependent manner [44]. HypoS caused a profound reduction in the development of RAD51 foci at all times, cell cycle phases and IR-doses measured, suggesting suppression of HR. Notably, this effect was reversible, and RAD51 foci rapidly returned, when cells were transferred to isotonic medium after a 1 h incubation in hypotonic medium. RPE cells also showed the previously reported suppression of HR at doses above 2 Gy [44]. The strong, reversible inhibition of RAD51 foci formation by HypoS was not specific to RPE cells and could be also detected in A549 cells exposed to different IR doses and analyzed by confocal microscopy using Cyclin B1 staining to identify cells in G_2_ (Figure S3c,d).

The above results document a stronger reduction in RAD51 foci as compared to γ-H2AX and 53BP1 foci in irradiated cells exposed to HypoS, which may be interpreted as a genuine suppression of HR rather than the result of chromatin decondensation that compromises foci development without affecting the underlying DDR response. To gather additional support for this interpretation, we employed a cell line, U2OS DR-GFP [46,47], harboring an integrated construct reporting the processing by HR of an I-*Sce*I-induced DSB through GFP expression that is quantified by FC.

Figure S4a summarizes the characteristics of the construct. Notably, Figure 5f and Figure S4b confirm that HypoS has a strong suppressive effect on HR, as less than a third of the control repair events were detected. This effect was not caused by incorrect GFP folding under hypotonic conditions, as the GFP signal from a GFP expression vector remained unchanged in hypotonic medium (Figure S4c). Furthermore, the suppression of HR after HypoS was not a consequence of shifts in cell cycle distribution, as the percentage of cells in G_2_/M-phase actually increased slightly in hypotonically treated cells, which would potentially produce more, rather than less, HR events (Figure S4d).

**Figure 6:**
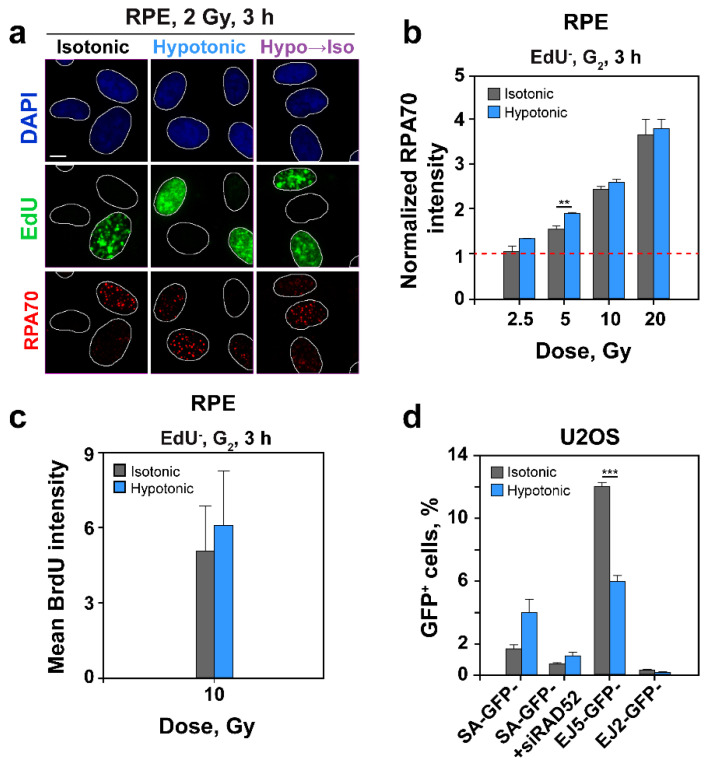
HypoS leaves DNA end-resection largely unchanged and enhances SSA. (**a**) Representative images of DAPI stained RPE nuclei showing EdU incorporation and RPA70 foci formation at 3 h post IR (2 Gy) in isotonically, hypotonically and transiently hypotonically (1 h) treated cells. Scale bar: 5 µm. (**b**) Arithmetic means of AF488 signal intensity for RPA70 measured by FC and gated for EdU^−^, G_2_-phase cells (as in Figure 2d) at 3 h post IR are normalized to the nonirradiated control values and plotted against IR dose. Means and SE are calculated from three experiments. (**c**) Arithmetic means of AF488 signal intensity for BrdU measured by FC and gated as in (**b**) for RPE cells maintained in isotonic (grey bar) and hypotonic medium (blue bar) for 3 h post 10 Gy IR. Means and errors are calculated from three independent experiments. (**d**) Percentage of GFP positive cells in U2OS SA-GFP, EJ5-GFP and EJ2-GFP reporter cell lines, 24 h after transfection with an I-*Sce*I expressing plasmid and treatment in hypotonic or isotonic medium. RAD52 knockdown in SA-GFP cells was carried out 24 h prior to treatment and in parallel with transfection of I-*Sce*I expressing plasmid. Grey bars: control cells in isotonic medium; blue bars: cells maintained in hypotonic medium. Means ± SE calculated from two to three independent experiments are shown. For all plots the significance of differences between individual measurements under isotonic and hypotonic conditions is indicated by the asterisk: ** *p* < 0.01 and *** *p* < 0.001.

A key step in the initiation of HR is DNA end-resection. We inquired whether the strong suppression of HR in cells exposed to HypoS is mediated by a suppression in resection. We employed the methods outline in Figure 2d and Figure 3c to carry out a cell cycle specific analysis of resection by measuring RPA70 signal using QIBC [39,40], or FC in cells co-stained with PI and EdU. Figure 6a shows examples of nuclei stained according to the required protocol, whereas Figure 6b shows the results of resection analysis in HypoS treated cells, focusing on EdU^−^, G_2_-phase cells exposed to different IR doses.

Surprisingly, and in sharp contrast to the strong suppression of HR following HypoS, resection was not detectably affected despite the perturbing effect on RPA70 foci visualization through chromatin decondensation. This result was confirmed by analysis of BrdU intensity levels post IR under hypotonic conditions (Figure 6c). Indeed, the small increase seen, which fails to reach statistical significance, may actually derive from the effect of HypoS in nonirradiated cells (see 0 Gy in Figure 2f). There is a trend for increased resection, preferably at low IR doses, but since the effect reached statistical significance only at 5 Gy, it is not elaborated here further.

Collectively, the above results demonstrate strong suppression of HR following HypoS, with resection remaining largely unaffected. We therefore hypothesized that other resection-dependent DSB repair pathways, such as SSA or alt-EJ, engage after HypoS as backup for suppressed HR.

### 2.6. HypoS Activates SSA to Compensate for HR Inhibition

The U2OS SA-GFP cell line reports SSA events, and it is evident that when cells were incubated in hypotonic medium, SSA rose 2.5 times over the controls (Figure 6d and Figure S5a,d). This increase is probably linked to the strong suppression observed in HR, as SSA is known to compensate for suppressed HR [48,49]. The function of SSA in this type of experiment is supported by the observation that knockdown of RAD52 (Figure S5e), an essential factor of SSA, reverses the increase observed in HypoS treated cells (Figure 6d). There was a small increase in G_2_/M-fraction (Figure S5f) in HypoS treated cells, but it was too small to cause the observed marked increase in SSA activity.

U2OS EJ5-GFP (Figure S5b) and U2OS EJ2-GFP (Figure S5c) cells report NHEJ and microhomology dependent alt-EJ, respectively. Both reporters showed a modest (up to 50%) inhibition, rather than potentiation, under hypotonic conditions (Figure 6d and Figure S5d).

### 2.7. HypoS Suppresses PCC Break Repair in G_2_, Increases Formation of Translocations and Radiosensitizes Cells to Killing

**Figure 7:**
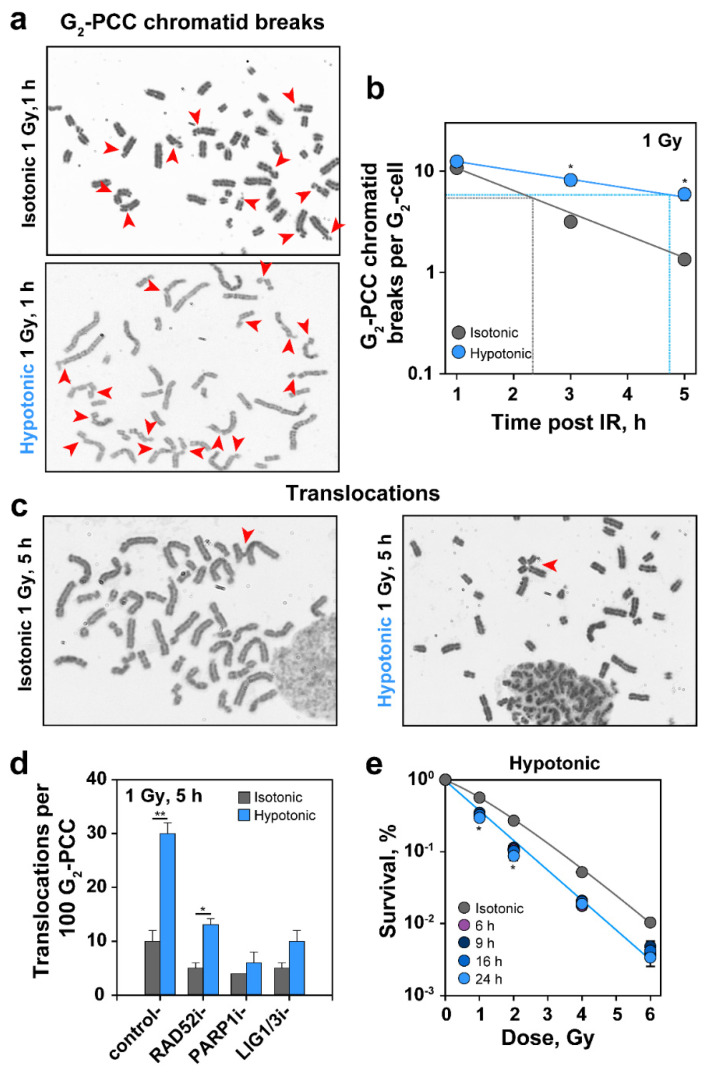
HypoS suppresses repair of interphase chromosome breaks, increases translocation formation and sensitizes cells to IR-induced killing. (**a**) RPE cells were incubated in isotonic or hypotonic medium after IR. G_2_-PCCs showing chromatid breaks (indicated by arrows) at 1 h after exposure to 1 Gy. (**b**) Kinetics of PCC break repair in cells exposed to 1 Gy and treated isotonically or hypotonically. Dotted lines indicate 50% chromatid break rejoining. Means ± SE are calculated from two experiments. Error bars are visible when larger than the symbols used. (**c**) Translocation formation in RPE cells exposed to 1 Gy and treated isotonically or hypotonically before processing for PCC. G_2_-PCCs at 5 h after exposure to 1 Gy showing translocations (indicated by arrows). (**d**) Quantification of translocations in cells treated with DMSO (control), RAD52 inhibitor (6-OH-DOPA, 10 µM), PARP1 inhibitor (PJ34, 5 µM), Ligase 1 and 3 inhibitor (L67, 50 µM), at 5 h after exposure to 1 Gy. Means ± SE are calculated from two independent experiments. (**e**) Clonogenic survival of isotonically and hypotonically treated RPE cells. Incubation in hypotonic medium after IR was carried out for 6, 9, 16 and 24 h (visible when the symbols are not overlapping). Means and SE are calculated from four determinations in two experiments. Error bars are visible when larger than the symbols used. For all plots, the significance of differences between individual measurements under isotonic and hypotonic conditions is indicated by the asterisk: * *p* < 0.05 and ** *p* < 0.01 (in (**e**) only if valid for all time points).

To further confirm the pronounced suppression of HR by HypoS in G_2_-phase cells, we took advantage of our recent observation that breaks forming in G_2_-phase prematurely-condensed chromosomes (PCC) after low IR doses required HR for their processing [50]. We reasoned that if the above shown suppression of HR in HypoS treated cells indeed occurs, then PCC processing in G_2_ should also be suppressed. Figure 7a shows representative cells in G_2_-phase (documented by the presence of fully replicated chromatids) in which PCC was induced 1 h after exposure to IR and transfer to isotonic or hypotonic medium. Note the chromosome breakage in both cells and the strongly elongated appearance of chromosomes in cells incubated in hypotonic medium, which confirms and extends the results shown in Figure 1d (see Figure S6a,b for length quantification).

Scoring of PCC breaks as a function of time confirms that incubation in hypotonic medium delayed their repair (Figure 7b). As a consequence, at 5 h after IR only about 50% of the induced chromatid breaks were repaired in cells exposed to HypoS, while isotonically kept controls repair in the same time interval almost 90% of induced chromatid breaks (see Figure S6c for representative images at different times of incubation in isotonic or hypotonic medium). This is in line with an increase in the rejoining half-time from 2.3 h to 4.7 h (dashed lines in Figure 7b). These results provide independent additional support to the notion that HypoS inhibits HR.

Since HR is the only error-free DSB repair pathway cells have at their disposal, its inhibition increases utilization of error-prone pathways. Such a pathway-switch is expected to increase the formation of chromosomal translocations. To test this postulate, we measured translocation formation in cells incubated in hypotonic medium. We analyzed cells in G_2_ after PCC induction, instead of cells at metaphase, to circumvent potential bias by the G_2_-checkpoint: long or permanent arrest of heavily damaged cells and selective release to mitosis of less damaged cells. Figure 7c shows representative examples and Figure 7d the results of such an analysis carried out 5 h after exposure to 1 Gy. Notably, HypoS caused a three-fold increase in translocation formation as compared to cells kept in isotonic medium. This result is fully in line with a shift in DSB processing to error-prone pathways, such as SSA or alt-EJ. Indeed, a specific RAD52 inhibitor strongly suppresses translocation formation. Inhibition of alt-EJ with a PARP1 inhibitor (PJ34), or a DNA Ligase 1/3 inhibitor (L67) also strongly suppressed translocation formation (Figure 7d).

Lastly, we measured the effect of HypoS on the survival of irradiated RPE cells. As a continuous (11 days) treatment in hypotonic medium was toxic, we tested treatments of 6, 9, 16 and 24 h before plating in normal cell culture medium. Figure 7e shows a modest sensitization to IR at all treatment times examined, in line with the effects on DDR and DSB repair described above. We note, however, that cell survival is measured here using exponentially growing cultures, which contain cells in all phases of the cell cycle, while the majority of the above-described DDR and DSB repair effects were specifically measured in G_2_-phase. Therefore, we expect a stronger radiosensitization in the G_2_-phase cells that represent less than 10% of the cell population tested here.

## 3. Discussion

### 3.1. General Physiological Effects of Hypos

At the organismal level, HypoS is an inflammatory stimulus increasing the production of cytokines such as IL-8 [51]. Extreme hypoosmotic conditions can induce cell burst, especially in combination with inhibitors of regulatory volume adaptations, which is exploited to kill cells during surgery in hepatocellular carcinoma or pancreatic cancer and to suppress thus peritoneal metastasis [52,53].

At the cellular level, homeostasis requires a precisely regulated ionic environment and osmolarity. Mammalian cells have developed sophisticated mechanisms to detect and respond to changes in osmolarity or ionic balance. Indeed, increases or decreases in extracellular solute concentration that cause sudden decreases or increases in cellular volume are promptly detected and counteracted by regulatory volume increases or decreases [33]. Such changes generate a multitude of effects on cell physiology, as they affect structure and function of biological macromolecules [34,35], deregulate ATP and calcium levels and cause a plethora of additional effects [54,55,56,57].

Here, we investigated effects of HypoS on DDR and DSB repair inspired by early work (discussed below) showing that post irradiation incubation of cells in an anisotonic environment causes pronounced radiosensitization to killing, suggesting a profound interference with repair processes. While the multitude of effects generated by anisotonic shocks complicates the establishment of cause-and-effect relationships, early work and the work presented here were designed on the working hypothesis that the effects observed on DSB repair predominantly derive from HypoS effects on global chromatin state and dynamics.

### 3.2. Effects of HypoS on Cell Cycle Progression and Cell Proliferation

As expected, RPE cells exposed to HypoS showed an increase in their volume within minutes. However, they adjusted to smaller volumes soon thereafter, owing to the inception of regulatory volume adaptations (Figure 1a). HypoS has been reported to reverse cytokinesis through disruption of myosin and F-actin [58], or through proteasome activation and subsequent degradation of cyclins and Cdks [59]. These effects show cell line specificity, depend on the degree of hypotonicity and generally inhibit cell proliferation. If the hypotonic environment is sustained, cells undergo apoptosis [60]. These reports are in line with our observation that RPE cells were unable to form colonies when maintained in hypotonic medium (Section 2.7). However, short-term, RPE cells adapted to HypoS and resumed proliferation with doubling times similar to those of cells maintained in isotonic medium (Figure 2a).

The transient effect of HypoS on cell proliferation was partly caused by a strong, transient drop in the progression of G_2_ cells to mitosis (Figure 2b) that bears similarities to IR induced G_2_-checkpoint in G_2_-irradiated cells. Notably, this response was ATM-independent despite ATM activation in HypoS exposed cells [41] (Figure 2c and Figure 3a,b), but partly ATR-dependent (Figure 2c). In line with ATR activation, increased levels of ssDNA by unknown mechanisms were detected in HypoS-treated cells by BrdU labelling (Figure 2f).

### 3.3. Effects of HypoS on Chromatin

Changes in tonicity have long been associated with changes in global chromatin state [28,61,62]. Indeed, we observed global chromatin decondensation after HypoS, evident by a decrease in CCP, as well as by an increase in the length of metaphase chromosomes, as well as of prematurely condensed G_2_-phase chromosomes (Figure 1b,d, Figure S1b,c, Figure 7a and Figure S6a,b). The observed chromosome elongation may derive from inhibitory HypoS effects on cohesins [63] or condensins. Indeed, depletion of condensins I or II leads to swollen or curly chromosomes, respectively, which is reminiscent to our observations [64].

The changes in global chromatin state in HypoS-treated cells were slower than those in cell volume, suggesting different mechanisms of detection and response. Notably, chromatin decondensation failed to show the expected markers of regulated chromatin organization accompanying transcription or replication, and was not associated with reductions in heterochromatin markers, such as H3K9me3 or HP1 (Figure 1e,f). The overall organization of DNA on nucleosomes seemed undisturbed, as MNase accessibility and digestion patterns remained unchanged (Figure 1g). Chromatin decondensation persisted for several hours, but recovered at 24 h and even showed signs of overcompensation (Figure 1b–d).

### 3.4. Effects of HypoS on DSB Signaling

Signaling at DSB starts with the activation of ATM that phosphorylates H2AX to generate γ-H2AX. This response is sensitive to chromatin organization [17]. Indeed, γ-H2AX accumulation is delayed in heterochromatin [43,65]. Vice-versa, artificial chromatin relaxation using HDAC inhibitors, or reduced levels of histone H1, enhances γ-H2AX spreading [66]. Our experiments show that in cells exposed to HypoS, both ATM activation, and γ-H2AX formation remained unchanged (Figure 3a,b, and Figure 4a,b), or were slightly enhanced, suggesting that the initial steps of DSB signaling remained active.

Notably, this conclusion is based on the analysis of ATM activation and γ-H2AX formation by Western blotting. Scoring of the corresponding foci by IF gave diverging results. Thus, pATM foci formation was strongly suppressed in irradiated HypoS exposed cells (Figure 3d,e), while γ-H2AX and 53BP1 foci formation was reduced to a lesser degree, mainly at high IR doses, more in S-and G_2_-phase and in a cell line-dependent manner (Figure 4c–e).

Although 53BP1 foci displayed a droplet-like behavior sensitive to hyperosmotic stress [67,68], the simplest explanation for this apparent discrepancy is the underlying change in chromatin state. We propose that changes in chromatin state of HypoS-exposed cells affects protein organization within the DDR focus and causes a dispersion effect that reduces its detectability by IF. Indeed, this effect may be construed as additional evidence for HypoS-induced chromatin decondensation. Despite the presumed lack of effect on 53BP1 response, we discuss below that HR is strongly suppressed by HypoS. We therefore suggest that HypoS disrupts the reciprocal connection between HR and 53BP1.

Finally, the results uncover inherent limitations when the effects on DDR of treatments altering chromatin structure are analyzed using as endpoint foci formation. In such cases, confirmation of the results using an independent method, such as Western blotting, may prove very helpful to the correct mechanistic interpretation of the results obtained.

### 3.5. HypoS Differentially Affects DSB Processing by Different Repair Pathways

DDR signaling ultimately regulates DSB repair, and it is generally thought that relaxation of chromatin not only facilitates detection and initial signaling but also accessibility of DSBs to proteins of all available repair pathways [69,70]. However, as already mentioned, local chromatin condensation is also observed at DSBs to increase the positional stability of the ends and facilitate repair [10,71,72,73]. The present work contributes to this discourse by analyzing the effect of HypoS-induced chromatin decondensation on the choice and thus the balance among available DSB repair pathways.

A key step in DSB repair pathway choice is the inception of 5’-end-resection that suppresses c-NHEJ and paves the way to one of the resection dependent pathways of DSB processing: HR, SSA or alt-EJ. Outside S- and G_2_-phase, c-NHEJ repairs the majority of DSBs [74], and even within these phases of the cell cycle, c-NHEJ dominates at high IR doses [44]. Our PFGE experiments show a small suppression of c-NHEJ under hypotonic conditions, mainly in the form of an increased contribution of the slow component of rejoining (Figure 5b and Figure S3b), which might reflect alt-EJ or SSA. A reporter assay also revealed a reduction by ~50% in the efficiency of this repair pathway (Figure 6d and Figure S5d) [75].

In contrast, and perhaps somewhat related, HypoS left unchanged, or only mildly enhanced, resection at DSBs (Figure 6b). Surprisingly, however, RAD51 foci formation was dramatically reduced, suggesting a strong inhibition of HR (Figure 5d,e). Owing to the uncertainties discussed above regarding foci detection in HypoS-treated cells, independent confirmation of this result with a functional assay was desirable. Indeed, an HR-reporter cell line confirmed that incubation in hypotonic medium caused a profound inhibition of HR [50] (Figure 5f and Figure S4b). This conclusion is further supported by analysis of chromatid break repair in G_2_-phase that is discussed in the following section. It is possible that chromatin decondensation in HypoS-treated cells increases the distance between sister chromatids, impairs homology search [69,76,77] and disrupts the normal evolution of HR, despite the presence of fully resected DNA ends.

Frequently, suppression of HR favors SSA [48,49], and the strong suppression of HR in HypoS-treated cells followed this trend, as it was linked to a 2.5-fold increase in RAD52-dependent SSA in the U2OS reporter cell line tested (Figure 6d and Figure S5d). SSA benefits from extensive resection [78], and possibly also global chromatin decondensation that makes DNA repeats, commonly located in compacted chromatin, more accessible. Indeed, a recent study shows that the interplay between 53BP1 and RNF169 regulates SSA by favoring or suppressing resection [79]. SSA is a highly mutagenic DSB repair pathway producing large deletions and, as our results show (Figure 7d, see discussion below), also chromosomal translocations [80,81].

Alt-EJ also benefits from resected DNA [82], but PFGE experiments showed no marked differences in its efficiency when chromatin was globally decondensed by HypoS treatment (Figure 5c). However, reporter cell lines detected suppression of alt-EJ in HypoS-treated cells (Figure 6d and Figure S5d), while analysis of chromosomal translocations suggested a strong increase (Figure 7d, see following section). We postulate that the endpoint-dependent effects of HypoS on alt-EJ, reflect differences in the engagement of this pathway at different doses of IR and for different subsets of DSBs. Inherent differences in assay sensitivity, as well as peculiarities of the biological parameters they employ as basis to estimate engagement, also may cause the observed differences in response. Finally, difficulties in discriminating among contributions by SSA and alt-EJ and the possibility that these two DSB repair pathways may even back up each other (see below), may contribute to the diverging results obtained.

Collectively, treatment of cells in hypotonic media generates highly differentiated responses with some DSB repair pathways being profoundly or mildly inhibited and others markedly activated. Such differentiated responses demonstrate that cells exposed to HypoS do not show a passive suppression of all their functions, as one might have predicted from the rather unspecific nature of the treatment, but an active response with differentiated inputs in different cellular functions. Indeed, the responses described here for HypoS are reminiscent to those recently published for the DNA repair/replication inhibitor araA, that also strongly suppresses HR while activating alt-EJ and SSA [83,84]. We anticipate that as a consequence, treatments in anisotonic media may prove useful in analyzing mechanistic contributions of chromatin dynamics to DDR, DSB repair and cell lethality.

### 3.6. Effects of HypoS on Chromatid Break Repair, Formation of Translocations and Cell Survival

HypoS strongly suppressed repair of breaks induced by IR in prematurely condensed chromosomes of G_2_-phase cells (Figure 7b). We recently reported that this form of repair reflects, at low doses of IR, almost exclusively the function of HR, and requires an active G_2_-checkpoint [50,85,86]. Therefore, the strong inhibition caused by HypoS treatment consolidates using an independent assay the notion that HypoS strongly inhibits HR.

Chromosomal translocations are hallmarks of cancer and markers of genomic instability and cell death [87,88,89]. The radiosensitization induced by HypoS was associated with a three-fold increase in translocations (Figure 7d) that was linked to the engagement of SSA and alt-EJ. Thus, HypoS radiosensitizes cells by suppressing error-free HR, while allowing the function of error-prone SSA and alt-EJ. The strong suppression in translocation formation caused by inhibition of alt-EJ or SSA factors, suggests interplay among these pathways and the ability to substitute for each other that warrants delineation through further work.

Changes in tonicity have long been associated with cell radiosensitization to IR-induced killing [28,61,62]. Several of these reports analyzed the profound radiosensitization induced by post irradiation hypertonic stress, and helped recognize that IR induces different forms of cellular damage, distinguishable from their repair kinetics ([32,90,91] reviewed in [29]). They also demonstrated that cell radiosensitivity can be strikingly modified by the post irradiation conditions employed, which led to the phenomenological definition of potentially lethal damage (PLD) [92]: A form of damage with undefined molecular identity, which depending on post irradiation conditions is either repaired allowing survival, or “fixed” to lethal damage (e.g., a translocation or chromosome deletion) causing cell death. Conditions delaying progression through the cell cycle support PLD repair and lead to an increase in cell survival, while changes in tonicity or treatment with DNA synthesis inhibitors, cause PLD fixation and radiosensitization [29].

Because PLD is phenomenologically defined, connections to specific forms of radiation damage and their repair pathways have remained elusive. However, six decades of accumulated evidence converge to the idea that molecularly, PLD represents DSBs. It follows that conditions favoring PLD repair support error-free and/or suppress error-prone DSB repair pathways, while post irradiation conditions causing PLD fixation inhibit error-free and/or activate error-prone DSB processing. In agreement and support of this notion, we recently reported that araA causes PLD fixation by strongly inhibiting HR [84], while enhancing at the same time, actively or passively, the function of alt-EJ [83]. The present work on HypoS follows along similar lines.

Collectively, the results on exposure to HypoS support the notion of DSB as the lesion underlying PLD and, for the specific case of G_2_-phase cells, the engagement of HR as the molecular process supporting PLD repair with SSA and alt-EJ promoting its fixation. More work will be required to elucidate the processes underpinning similar responses in other phases of the cell cycle.

In summary, our results show that HypoS causes chromatin decompaction that is not linked to standard histone modifications of regulated chromatin decondensation. HypoS allows normal development of DDR but compromises the organization of the associated proteins as foci. Strikingly, HypoS markedly suppresses HR without affecting resection, and favors SSA as a result. It exerts a relatively small effect on c-NHEJ and alt-EJ. These pronounced shifts in DSB repair pathway engagement are accompanied by a marked decrease in G_2_-phase chromatid break repair and by large increases in chromosomal translocations that ultimately sensitize cells to IR-induced killing. These observations substantially expand our understanding of key facets of the effects of anisotonic environments on DDR and DSB repair and generate interesting connections to PLD repair.

## 4. Materials and Methods

### 4.1. Cell Culture and Cell Volume Measurements

Cells were maintained at 37 °C in an atmosphere of 5% CO_2_ and 95% air. Human RPE-1 hTert (henceforth RPE) cells were grown in Dulbecco’s modified minimum essential medium (DMEM) supplemented with 10% fetal bovine serum (FBS) and antibiotics. U2OS DR-GFP, U2OS SA-GFP, U2OS EJ5-GFP and U2OS EJ2-GFP [46,47,93] and A549 (human lung epithelial carcinoma) cells were grown in McCoy’s 5A medium supplemented with 10% FBS and antibiotics. Human 82-6 hTert cells were grown in minimum essential medium (MEM) supplemented with 10% FBS, 1% nonessential amino acids (NEAA) and antibiotics. All cell lines were passaged thrice a week.

For hypotonic treatment, cell growth medium was diluted by adding an equal volume of double-distilled H_2_O (henceforth “hypotonic medium”), while in control cultures an equal volume of a 150 mM NaCl solution was added (henceforth “isotonic medium”). Cell volume measurements were performed using a Multisizer Z2 cell counter (Beckman Coulter, Krefeld, Germany) under hypotonic (standard cell counting solution diluted 1:1 with double-distilled H_2_O) or isotonic (standard cell counting solution) conditions, respectively. When cells were irradiated, transfer in hypotonic or isotonic medium was always carried out immediately after irradiation. Standard protocols were employed to determine clonogenic survival.

### 4.2. Radiation Exposure

Irradiations were carried out using an X-ray machine (GE Healthcare, Buckinghamshire, UK) operating at 320 kV, 12.5 mA with a 1.65 mm Al filter at a distance of 50, 66 or 75 cm and a dose rate of 3.5, 2.2 or 1.7 Gy/min, respectively. Dosimetry was performed with a PTW and/or a chemical dosimeter, which were used to calibrate an infield ionization monitor.

### 4.3. Inhibitors

The following inhibitors were used at the indicated final concentrations as described previously [39]: 8-(4-Dibenzothienyl)-2-(4-morpholinyl)-4H-1-benzopyran-4-one (NU7441, DNA-PKcsi, Haoyuan ChemExpress, Shanghai, China), a specific DNA-PKcs inhibitor at 5 µM. 2-Morpholin-4-yl-6-thianthren-1-yl-pyran-4-one (KU55933, ATMi, Haoyuan ChemExpress), a specific ATM inhibitor at 10 µM. 3-Amino-6-[4-(methylsulfonyl)phenyl]-*N*-phenyl-2-pyrazinecarboxamide (VE-821, ATRi, Haoyuan ChemExpress) a specific ATR inhibitor at 5 µM. An RAD52 inhibitor (6-OH-DOPA, RAD52i [94]) at 10 µM. The PARP inhibitor PJ34 (Calbiochem^®^, Merck KGaA, Darmstadt, Germany) at 5 µM. Ligase 1 and Ligase 3 inhibitor L67 (LIG1/3i) [95] at 50 µM. RAD52i was dissolved in 1 N HCl; all other inhibitors were dissolved in DMSO (Sigma-Aldrich^®^, Merck KGaA, Darmstadt, Germany). Inhibitors were added 1 h before IR and were maintained until collection of cells for analysis.

### 4.4. Micrococcal Nuclease (MNase) Assay

Exponentially growing cells were incubated in isotonic or hypotonic medium as indicated. Cells were collected by trypsinization (without EDTA) and lysed in lysis buffer (50 mM Tris-HCl pH 8, 5 mM CaCl_2_, protease inhibitors (1:100, Halt^TM^, Thermo Fisher Scientific, Waltham, MA, USA), 1% IGEPAL CA-630 (Sigma-Aldrich^®^, Merck KGaA, Darmstadt, Germany) (50 µL per 0.5 × 10^6^ cells) for 30 min on ice. Digestion was performed with 10 U MNase solution (Thermo Fisher Scientific, Waltham, MA, USA) for 10 min at 37 °C and stopped by adding an equal volume of stop buffer (400 mM NaCl, 40 mM EGTA (Sigma-Aldrich^®^, Merck KGaA, Darmstadt, Germany), 2% SDS (Carl Roth GmbH + Co. KG, Karlsruhe, Germany), 2.5% Proteinase K (Macherey-Nagel, NucleoSpin Tissue Kit). Samples were purified using Macherey-Nagel NucleoSpin Gel and PCR clean-up kit according to manufacturer’s instructions. Entire eluate was run in a 1% agarose gel cast with ethidium bromide (EtBr), in TAE buffer (40 mM Tris-HCl, 20 mM acetic acid, 1 mM EDTA) at 70 V for 2 h. Gels were scanned and digitized on a Typhoon Imager (GE Healthcare, Buckinghamshire, UK).

### 4.5. Pulsed-Field Gel Electrophoresis (PFGE)

PFGE was used to assess induction and repair of DSBs as previously described [96,97]. EtBr stained PFGE gels were scanned in a Typhoon Imager (GE Healthcare, Buckinghamshire, UK) and the fraction of DNA released (FDR) from the plug into the lane quantified by ImageQuant 5.2 software (GE Healthcare, Buckinghamshire, UK). Dose response curves were plotted as FDR values versus IR dose. Fitted lines were used to calculate the equivalent Gy-dose values (DEQ) for each FDR measured at a given repair time point; repair kinetics are given as plots of DEQ versus time.

### 4.6. Indirect Immunofluorescence and Image Analysis

For immunofluorescence (IF) analysis, cells were grown on coverslips and treated with isotonic or hypotonic medium. Sample preparation was performed as described previously [39]. The following primary antibodies were used: anti-γ-H2AX [3F2] mouse monoclonal antibody (Abcam, Cambridge, UK), anti-53BP1 rabbit polyclonal antibody (Santa Cruz Biotechnology, Inc., Heidelberg, Germany), anti-RAD51 mouse monoclonal antibody (GeneTex, Inc., Irvine, CA, USA), anti-ATM-pS1981 mouse monoclonal antibody (Cell Signaling Technology Europe, Frankfurt a. M., Germany), anti-RPA70B mouse monoclonal antibody [98] and an anti-cyclin B1 rabbit polyclonal antibody H-433 (Santa Cruz Biotechnology, Inc., Heidelberg, Germany). Primary antibodies were incubated for 1.5 h and removed by three PBS washing steps (5 min) to add the anti-mouse IgG or anti-rabbit IgG secondary antibodies (Alexa Fluor488-conjugated, Alexa Fluor568-conjugated or Alexa Fluor647-conjugated) for 1 h (Life Technologies, Thermo Fisher Scientific, Waltham, MA, USA) according to manufacturer’s instructions. When EdU labeling was applied, slides were also processed with an EdU staining cocktail (1 M Tris-HCl pH 7.4, 10 mM CuSO_4_, 1 mM azide dye (AlexaFluor647 or 6-FAM) and 500 mM ascorbic acid in PBS). Finally, cells were counterstained with DAPI (50 or 200 ng/mL in PBS) for 10 min at room temperature (RT) and embedded in Prolong Gold Antifade mounting media (Thermo Fisher Scientific, Waltham, MA, USA). Samples were either scanned on a Leica TCS-SP5 confocal microscope, or on an AxioScan.Z1 imaging platform (Zeiss, Oberkochen, Germany) for cell cycle dependent evaluation of IRIF by QIBC as described before [44]. Sequential scanning was employed to exclude spillover from different channels.

For analysis of the captured three-dimensional image stacks, the module of the Imaris 8.0–9.3 software (Bitplane, Belfast, UK) “Spots and Split Spots” was used to score foci numbers. Alternatively, the “Cell module” of the same software was utilized. The grayscale value thresholds for the separation of signal and background were kept constant in different experiments with the same antibody-batch to ensure comparability between data sets. Only objects with a minimum diameter of 0.5 µm after thresholding were counted as foci. For every dose and time point, at least 150 cells were quantified. When Cyclin B1 was used to identify G_2_-phase cells, only cells with clearly visible Cyclin B1 staining in the cytoplasm were included in the analysis. For cell cycle specific analysis of DSB repair foci in G_1_-phase, EdU^+^, G_2_-phase and EdU^−^, G_2_-phase cells, data obtained using the Imaris software were processed by the open source graphic software package, Orange.

The CCP was determined as described in [38]. Nuclei were imaged in a confocal microscope at a resolution of 1024 × 1024 pixels per image. Per time point 132–275 cells were scanned. Using ImageJ software, images in grayscale containing single nuclei were generated using the macro described under Supplementary Materials. All calculations were performed using MatLab after adaptation to our purposes of the codes described by Irianto et al. [38] (see Supplementary Material for codes and Figure S1a for processing steps).

### 4.7. Flow Cytometry (FC) Analysis

Three-parametric FC was used to measure DNA end-resection by quantification of BrdU or RPA70-signal in G_2_-phase cells. For BrdU incorporation, cells were incubated with 10 µM BrdU 24 h prior to EdU labeling. Cells were washed with PBS to avoid competition of BrdU and EdU before pulse-labeling with EdU for 30 min prior to IR that was used to label cells in S-phase at the time of radiation exposure. At different time points after IR, cells were permeabilized in cold 1X PBS containing 0.2% Triton X-100 for 2 min on ice. Fixation in 2% PFA solution for 15 min at room temperature followed and cells were blocked in PBG solution overnight at 4 °C. Cells were incubated with a specific BrdU antibody (anti-BrdU mouse monoclonal antibody, Becton Dickinson, Heidelberg, Germany) or a RPA70-antibody (see Section 4.6), diluted in PBG, for 1.5 h at room temperature. After washing with PBS, labeling was performed with secondary antibodies and the EdU staining cocktail as described above (Section 4.6). Finally, DNA was stained with propidium iodide (PI) for 30 min at 37 °C. Analysis was carried out in a flow cytometer (Gallios, Beckman Coulter, Krefeld, Germany) and quantified using available software (Kaluza 1.3–2.1, Beckman Coulter, Krefeld, Germany).

To determine the mitotic index (MI) and H3K9me3 and HP1 levels, two-parametric FC was employed. For MI determinations, cells were fixed in ice-cold 70% ethanol (EtOH) and permeabilized in ice-cold PBS containing 0.25% Triton X-100. For H3K9me3 and HP1 analysis, cells were processed as described above. Cell pellets were blocked in PBG overnight at 4 °C prior to incubation for 1.5 h at room temperature with an anti-HistoneH3-pS10 specific antibody (rabbit polyclonal, Abcam, Cambridge, UK), an anti-HistoneH3K9me3 rabbit polyclonal antibody (Abcam, Cambridge, UK) or an anti-HP1 (E-6) mouse monoclonal antibody (Santa Cruz Biotechnology, Inc., Heidelberg, Germany) diluted in PBG. After washing with PBS, cells were incubated with secondary antibody, conjugated with AlexaFluor488 (Life Technologies, Thermo Fisher Scientific, Waltham, MA, USA) and DNA was stained with PI for 30 min at 37 °C. Analysis was carried out in a flow cytometer (Gallios, Beckman Coulter, Krefeld, Germany) equipped with the appropriate software (Kaluza 1.3–2.1, Beckman Coulter, Krefeld, Germany); for MI determinations, gating was applied to select H3pS10 positive events in the G_2_-compartment that represent mitotic cells. The mitotic index (MI) was determined as the fraction of cells in mitosis and is shown normalized to the MI of untreated controls.

### 4.8. Protein Extraction, SDS-PAGE and Western Blot Analysis

Cells were collected by trypsinization, washed with PBS and incubated in ice cold RIPA buffer (Pierce^®^, Thermo Fisher Scientific, Waltham, MA, USA) containing protease inhibitors (1:100, Halt^TM^, Thermo Fisher Scientific, Waltham, MA, USA) and/or phosphatase inhibitor cocktail set IV (1:100, Merck Millipore, Merck KGaA, Darmstadt, Germany) for 30 min on ice. Cell lysis was expedited with three pulses of ultra-sonication. Cell lysates were centrifuged at 12,000 rpm for 15 min at 4 °C and the supernatants (RIPA whole-cell protein extract) collected for further analysis. The protein concentration was determined by the Bradford assay. Standard protocols for SDS-PAGE and immunoblotting were employed. Typically, 50–70 µg of total protein extracts were loaded on each lane. The primary antibodies used were: anti-RAD52 mouse monoclonal antibody (Santa Cruz Biotechnology, Inc., Heidelberg, Germany), anti-γ-H2AX [3F2] mouse monoclonal antibody (Abcam, Cambridge, UK), anti-phospho-ATM (Ser1981) (D6H9) rabbit monoclonal antibody (Cell Signaling Technology Europe, Frankfurt a. M., Germany), anti-KAP1-pS824 rabbit polyclonal antibody (Bethyl Laboratories, Montgomery, TX, USA) and anti-GAPDH (GA1R) mouse monoclonal antibody (UBPBio, Dallas, TX, USA). The secondary antibodies were anti-mouse and anti-rabbit IgG conjugated with IRDye680 and IRDye800 (LI-COR Biosciences, Lincoln, NE, USA). Immunoblots were scanned on the Odyssey infrared scanner (LI-COR Biosciences, Lincoln, NE, USA).

### 4.9. RNA Interference

To deplete RAD52, knockdown experiments were carried out using the following specific siRNAs: Negative control (siNC) (UUCUCCGAACGUGUCACGU), RAD52 (GGCCCAGAAUACAUAAGUATT). The siRNA was delivered to the cells by nucleofection, using the Nucleofector 2D device (Lonza Bioscience, Basel, Switzerland). The level of knockdown was assessed by quantitating protein levels using western blot analysis 24 h after nucleofection.

### 4.10. Analysis of HR, c-NHEJ, alt-EJ and SSA Function Using GFP Reporter Cell Lines

U2OS-GFP cell lines reporting the processing of I-*Sce*I-induced DSBs by a specific repair pathway were employed to quantify the effects of different treatments [46,47,93]. The DR-GFP cell line reports the function of HR, the EJ5-GFP cell line reports the function of c-NHEJ, the EJ2-GFP cell line reports the function alt-EJ and the SA-GFP cell line reports the function of SSA. For knockdown experiments, transfection with siRNA was performed 24 h prior and at the same time as transfection with the I-*Sce*I expressing plasmid.

### 4.11. Cytogenetics

For metaphase analysis, exponentially growing cells were incubated at 37 °C in isotonic or hypotonic medium until collection (1–24 h). Metaphases were accumulated by adding 0.1 µg/mL Colcemid (L-6221, Biochrom AG, Merck Millipore, Merck KGaA, Darmstadt, Germany), 30 min before collection, for each time point and processed as described earlier [99]. Metaphase spreads were fixed in 2% PFA solution, counterstained with DAPI (50 ng/mL in PBS) for 10 min at room temperature and mounted in Prolong-Antifade mounting media. A MetaSystems station with a microscope (AxioImager.Z2, Zeiss, Oberkochen, Germany) and automated image capture and analysis capabilities were used for imaging chromosomes. For length quantification of metaphase chromosomes Adobe Photoshop CS5 ruler tool was used. For chromosome break analysis using G_2_-phase premature chromosome condensation (G_2_-PCC), exponentially growing cells were incubated post IR at 37 °C in isotonic or hypotonic medium. To induce G_2_-PCC, 50 nM calyculin-A (LC Laboratories, Biomol, Hamburg, Germany) was added for 1 h before harvesting at the respective time point. The time of calyculin-A treatment was not included in the indicated repair times. Approximately 50 G_2_-PCCs were scored for each experimental time point from two independent experiments and excess PCC fragments were calculated. Analysis was carried out using a bright field microscope (Leica Microsystems DM RBE, Wetzlar, Germany) connected to a camera.

### 4.12. Statistical Analysis

Graphs were created in SigmaPlot 12.5 and 14. Statistical significance was determined using the Student’s t-test routine available in SigmaPlot 12.5 and 14. Statisitical significance was indicated as * *p* < 0.05, ** *p* < 0.01, or *** *p* < 0.001.

## Supplementary Materials

The supplementary materials are available online at https://www.mdpi.com/article/10.3390/ijms222010957/s1.
