# Supplementary material for: Disruption of Chromatin Dynamics by Hypotonic Stress Suppresses HR and Shifts DSB Processing to Error-Prone SSA
Source: Int J Mol Sci. 2021 Oct 11;22(20):10957. doi: 10.3390/ijms222010957 (PMC8535785; doi:10.3390/ijms222010957)
Supplement: Supplementary file 1 [file ijms-22-10957-s001.zip › ijms-1373233-supplementary.pdf]

Supplementary Material

HypoS decondenses chromatin.

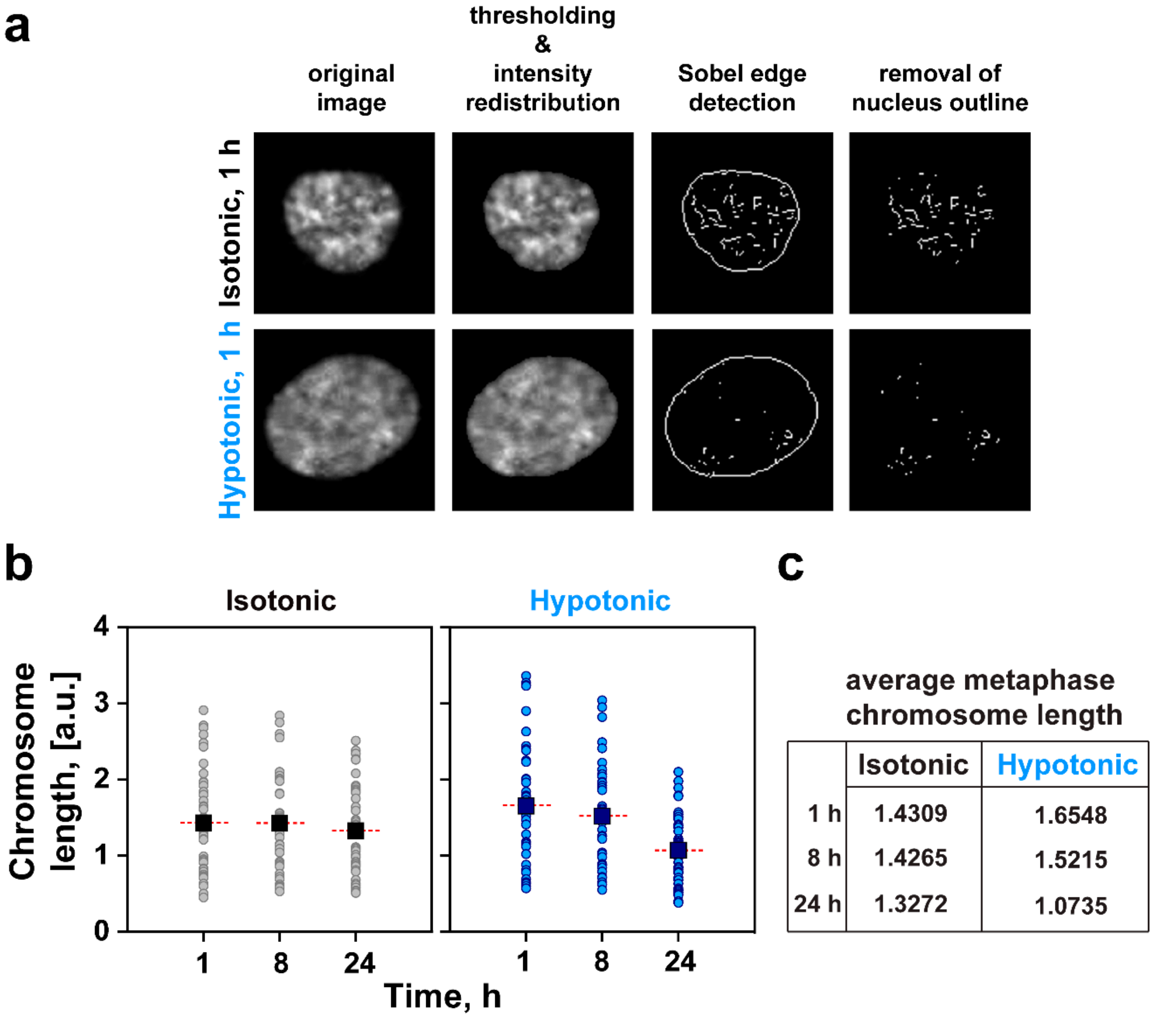

c

average metaphase chromosome length

	Isotonic	Hypotonic
1 h	1.4309	1.6548
8 h	1.4265	1.5215
24 h	1.3272	1.0735

Figure S1. (a) Intermediate processing steps of CCP determination using the codes by Irianto et al. [38]. RPE nuclei at different stages of processing as outlined in Supplementary Materials. (b) Dot plots depicting metaphase chromosome lengths determined using the Adobe Photoshop CS5 ruler tool in the metaphase chromosomes shown in Figure 1d. Squares and red, dashed lines represent individual chromosome lengths and their mean value, respectively, following incubation in hypotonic medium for the indicated times. (c) Mean values of results in b.

DDR foci formation under HypoS in different cell lines.

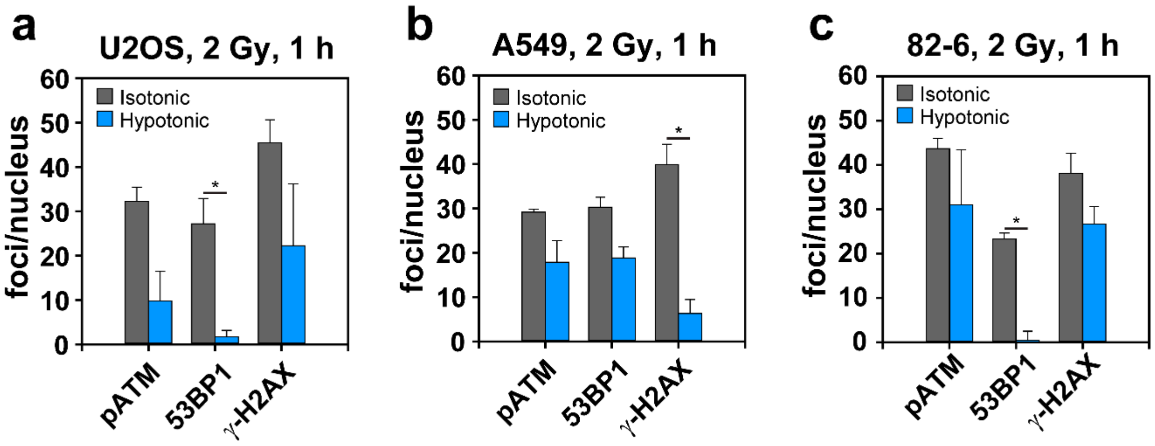

Figure S2. (a, b and c) pATM, γ-H2AX and 53BP1 foci analysis in G_2-phase (EdU^-) cells exposed to 2 Gy and subject to HypoS for 1 h: (a) U2OS cells, (b) A549 cells and (c) 82-6 hTert cells. Means and SE from two experiments are shown. The significance of differences between isotonic and hypotonic treatment is indicated by *$p<0.05$.

DSB repair under HypoS.

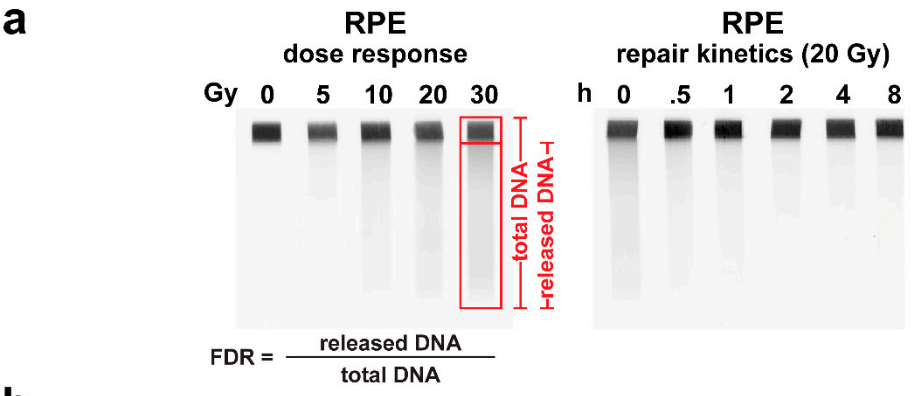

b

treatment	plot equation	$t_{50,slow}$	FDR_{slow}
Isotonic treatment	$f(t) = 20e^{-3.4721t} + 2.4935e^{-0.2165t}$	192 min	11 %
Hypotonic treatment	$f(t) = 20e^{-2.2668t} + 4.3627e^{-0.1723t}$	241 min	18 %
Isotonic treatment + DNA-PKcsi	$f(t) = 20e^{-1.108t} + 8.7245e^{-0.0224t}$	31 h	30 %
Hypotonic treatment + DNA-PKcsi	$f(t) = 20e^{-1.3191t} + 8.0334e^{-0.0147t}$	47 h	29 %

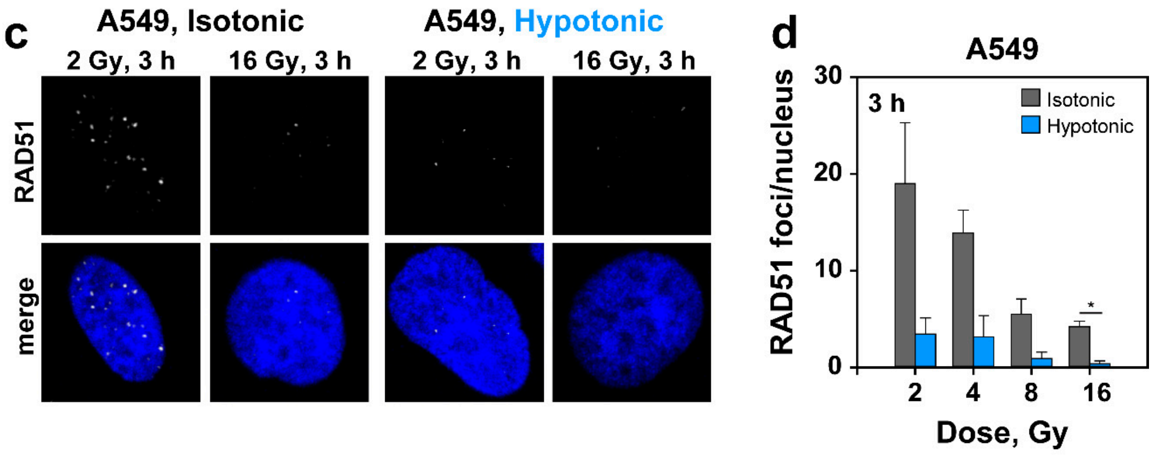

Figure S3. (a) Representative images of EtBr stained PFGE gels showing dose response (left) and repair kinetics (right) in RPE cells. FDR is calculated by dividing the signal from DNA released from the well into the lane of the gel by that of total DNA in the sample, as indicated. (b) Half-times for the slow component (t_{50},slow) of DSB repair, as well as the fraction of DSBs repaired by this component (FDR_{slow}), calculated as described earlier [97]. (c) Representative images of RAD51 foci formation in A549 cells at 3 h after exposure to 2 or 16 Gy of IR and incubation in isotonic or hypotonic medium. (d) Dose response of RAD51 foci formation in A549 cells at 3 h post IR after incubation in isotonic or hypotonic medium. The means and SEs from two independent experiments are shown. Statistical significance is indicated by *p<0.05.

Reporter assay DR-GFP under HypoS.

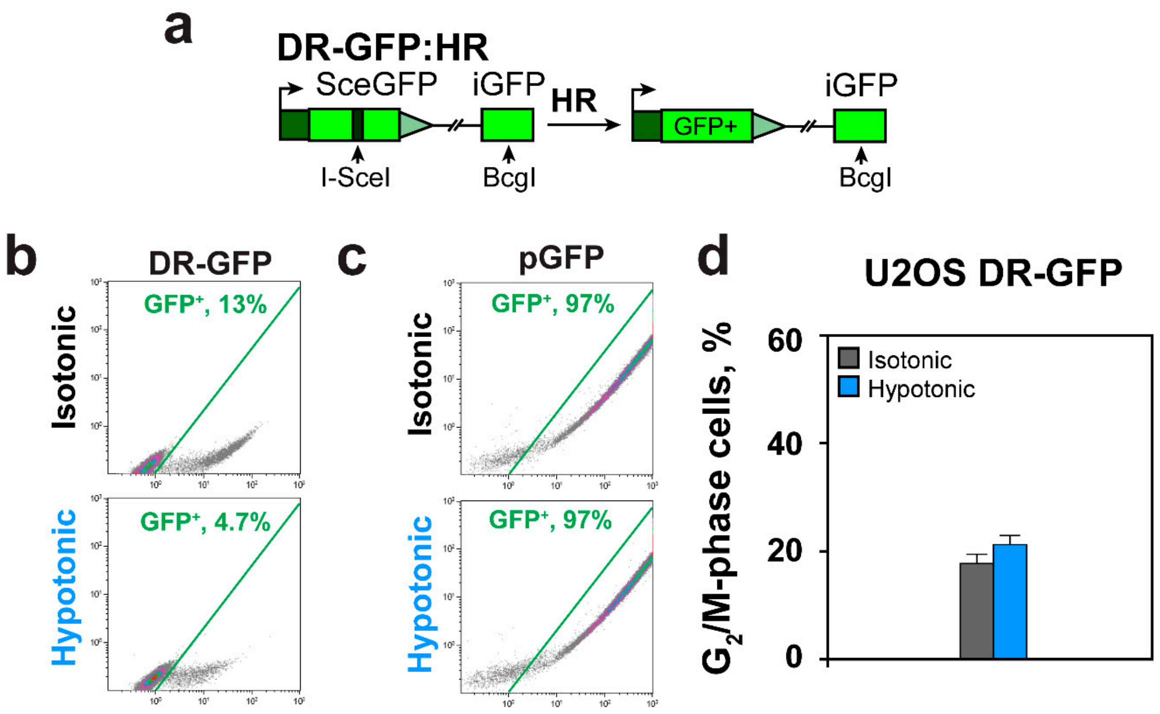

Figure S4. (a) Schematic drawing of the DR-GFP reporter construct for DSB repair by HR [46, 93]. (b) GFP signal measured by FC in U2OS DR-GFP cells after transfection by nucleofection of an I-*Sce*I expressing plasmid. Analysis is carried out 24 h after transfection and HypoS initiated 2.5 h after transfection. (c) GFP signal measured by FC in U2OS DR-GFP cells, 24 h after transfection by nucleofection with an pEGFP-N1 plasmid (other details as in b). (d) Percentage in G_2/M-phase of U2OS DR-GFP reporter cells, 24 h after incubation in isotonic or hypotonic medium (other details in b). Means and SEs from four experiments are shown. The p-value here fails to reach statistical significance.

Reporter assays in U2OS cells under HypoS.

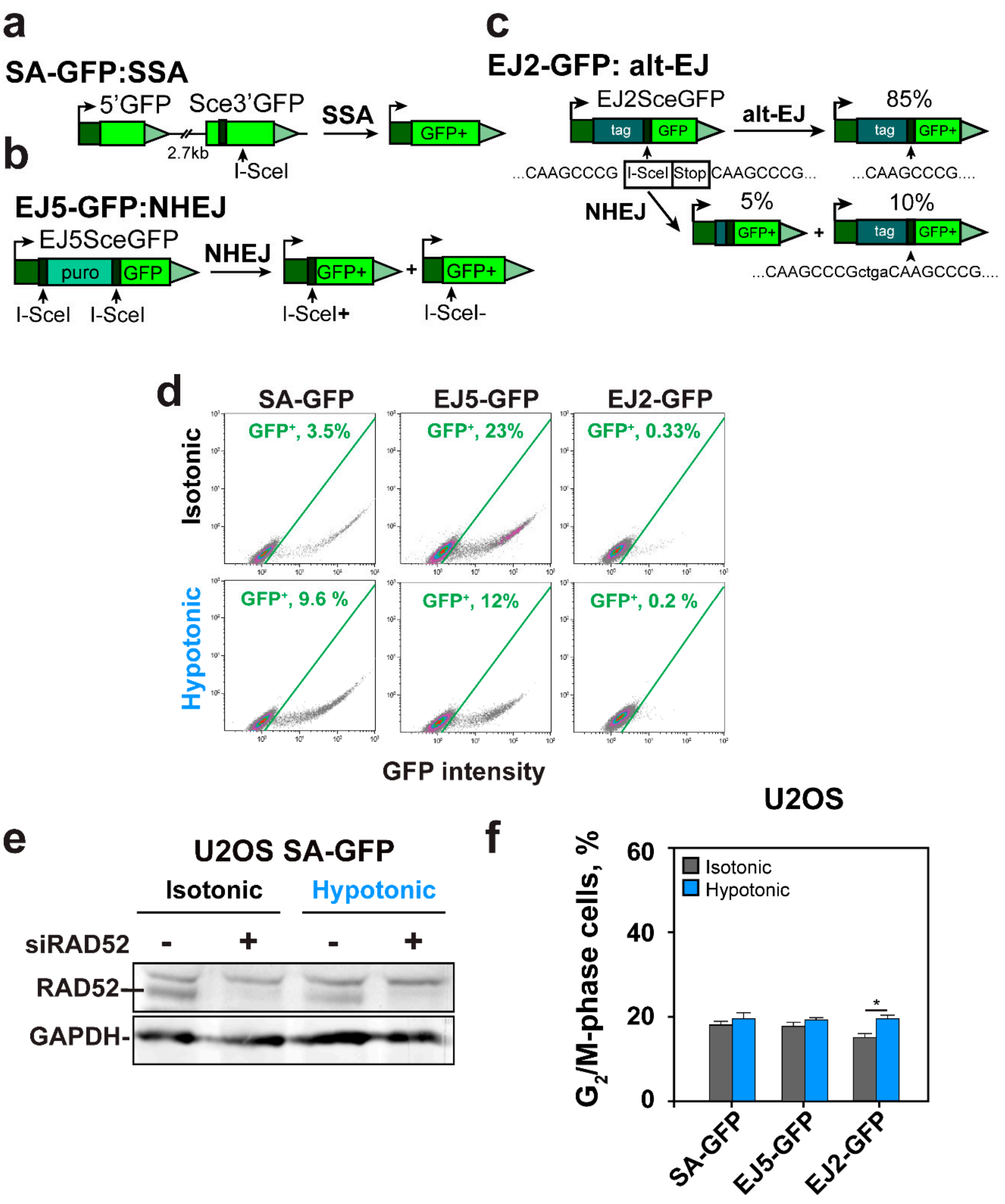

Figure S5. (a-c) Schematic drawing of the U2OS reporter constructs for the different DSB repair pathways as indicated [46,93]. (d) As in Figure S4b for U2OS SA-GFP, EJ5-GFP and EJ2-GFP cells. (e) Western blot of U2OS SA-GFP cells treated isotonically or hypotonically in combination with siRNA

mediated knockdown of RAD52. (f) As in Figure S4d for U2OS SA-GFP, EJ5-GFP and EJ2-GFP. Statistical significance is indicated by *p<0.05.

G_2-PCCs under HypoS.

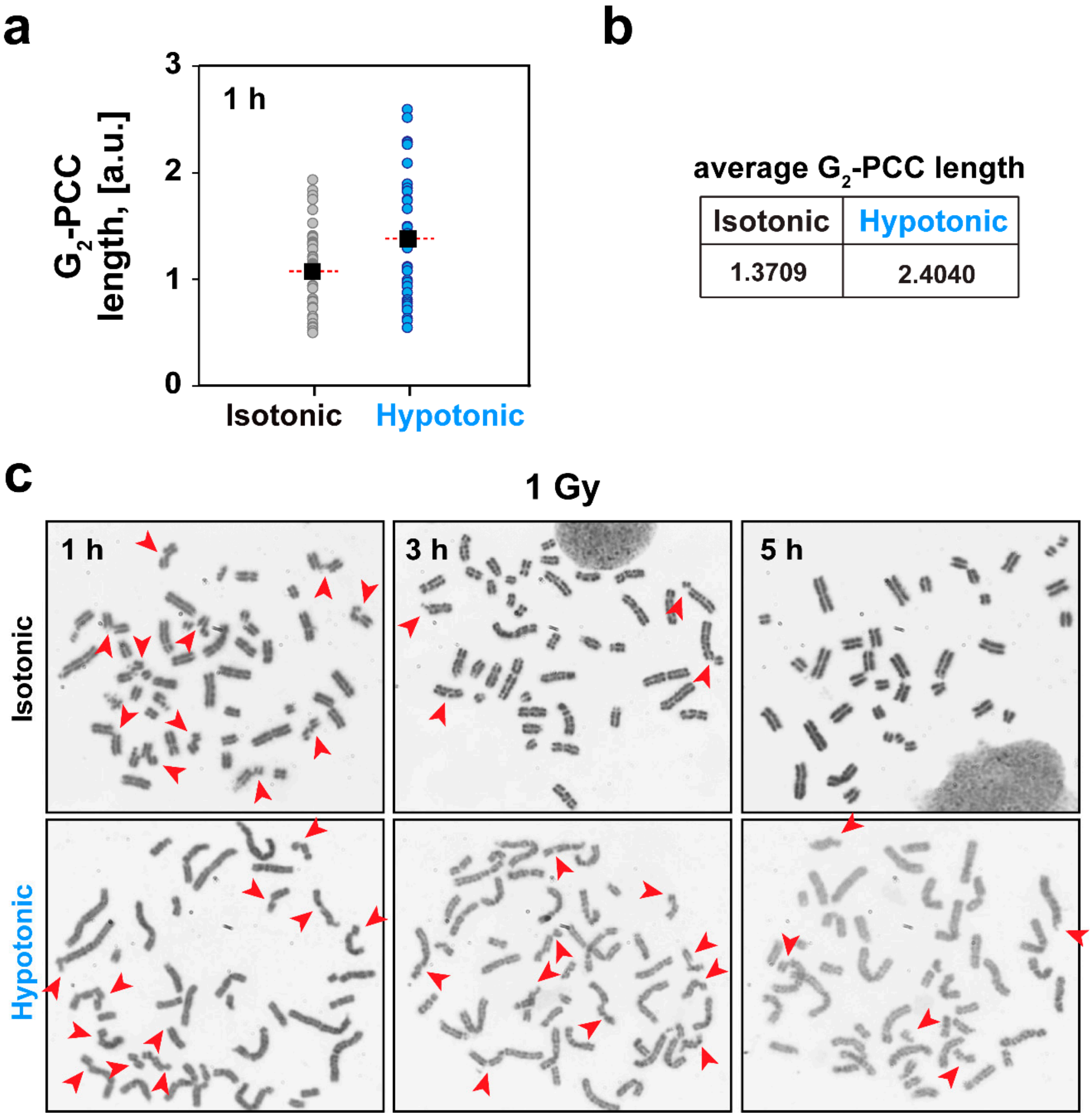

Figure S6. (a) Same as in Figure S1b for G_2-PCCs of RPE cells treated in isotonic or hypotonic medium for 1 h. Circles represent chromosome lengths and squares and red dashed lines mean values of chromosome lengths. (b) Mean values of results in a. (c) Representative PCC images showing chromatid breaks (indicated by arrows) in RPE cells at the indicated times after exposure to hypotonic or isotonic medium following exposure to 1 Gy of IR.

Supplementary information:

<u>CCP analysis (adapted from Irianto et al. 2014 [38])</u>

Image processing by ImageJ:

For generating single nuclei images out of microscopy images, lif-files are imported to ImageJ, maximum intensity images in grey scale are generated for each stack and from those single nuclei images using the following macro code are produced.

```
requires("1.51w");
dir = getDirectory("Where to save the nuclei");
setBatchMode( true );
orig = getImageID();
img = split(getTitle(), ".");
run("Duplicate...", " ");
nme = getTitle();
run("Split Channels");
close(nme+" (green)");
close(nme+" (red)");
selectImage(nme+" (blue)");
blue = getImageID();
setAutoThreshold("Mean dark");
setOption("BlackBackground", false);
run("Convert to Mask");
run("Median...", "radius=1");
run("Fill Holes");
run("Watershed");
imageCalculator("Multiply create 32-bit", blue, orig);
rslt = getImageID();
selectImage(blue);
close();
selectImage(orig);
close();
selectImage(rslt);
run("8-bit");
setAutoThreshold("Mean");
run("Analyze Particles...", "size=0-10000 show=Nothing exclude add");
resetThreshold();
run("Invert LUT");
cnt = roiManager("count");
for ( i=0; i<cnt; i++ ) {
        roiManager("select", i);
        run("Duplicate...", " ");
        run("Clear Outside");
        run("Remove Overlay");
        save( dir+img[0]+"_nuc-"+(i+1)+".tif" );
        close();}
setBatchMode( false );
exit();
```

MatLab codes for calculating CCP:

Apply Thresh:

```
function [IThresh] = ApplyThresh(I,T)
```

```
%%%%%%%%%%%%%%%%%%%%%%%%%%%%%%%%%%%%%%%%%%%%%%%%%%%%%%%%%%%%%%%%
%%%%%%%%%%%%%%%%
%This function receive the image to be thresholded and the threshold value.
%This function then produce a thresholded image.
%I: image to be thresholded
%T: threshold value
%%%%%%%%%%%%%%%%%%%%%%%%%%%%%%%%%%%%%%%%%%%%%%%%%%%%%%%%%%%%%%%%
%%%%%%%%%%%%%%%%

clear row column int S sizerow sizecolumn R C

[row,column,int] = find(I>T);
S = length(row);
[sizerow,sizecolumn] = size(I);
IThresh = zeros(sizerow,sizecolumn);
for i = 1:S
   R = row(i,1);
   C = column(i,1);
   IThresh(R,C) = 1;
end
```

Extract Image:

```
function [IExtract] = ExtractImage(I,In)

%%%%%%%%%%%%%%%%%%%%%%%%%%%%%%%%%%%%%%%%%%%%%%%%%%%%%%%%%%%%%%%%
%%%%%%%%%%%%%%%%
%This function receive the image to be extracted to a black background and
%the thresholded image (having the pixels locating where the target is).
%This function then produce a target image with black background.
%I:     target image
%In:    thresholded image
%%%%%%%%%%%%%%%%%%%%%%%%%%%%%%%%%%%%%%%%%%%%%%%%%%%%%%%%%%%%%%%%
%%%%%%%%%%%%%%%%

clear row column int S sizerow sizecolumn R C

[row,column,int] = find(In>0);
S = length(row);
[sizerow,sizecolumn] = size(In);
IExtract = zeros(sizerow,sizecolumn);
for i = 1:S
   R = row(i,1);
   C = column(i,1);
   IExtract(R,C) = I(R,C);
end
```

GeneratingPrint:

```
function [PrintList] = GeneratingPrint(PrintIndex,q,PrintNameList,I2,I3,I4,I5,I6,I7,A1,I8,I9,I10,I11,I12,I13)

%%%%%%%%%%%%%%%%%%%%%%%%%%%%%%%%%%%%%%%%%%%%%%%%%%%%%%%%%%%%%%%%
%%%%%%%%%%%%%%%%
%This function receive an Index, which show the chosen matrices, and the
%q-th image being processed.
%This function then produce prints of the chosen matrices
%PrintIndex:    The index of chosen matrices (m x 1)
%q:             The q-th image being processed
%%%%%%%%%%%%%%%%%%%%%%%%%%%%%%%%%%%%%%%%%%%%%%%%%%%%%%%%%%%%%%%%
%%%%%%%%%%%%%%%%

Index = find(PrintIndex>0);
Cond = isempty(Index);

if Cond == 0
   for i = 1:size(Index,1)
```

```
        ImageChosen = Index(i,1);
        if ImageChosen == 1
            imwrite(I2,PrintNameList(q,i).name,'tif');
        elseif ImageChosen == 2
            imwrite(I3,PrintNameList(q,i).name,'tif');
        elseif ImageChosen == 3
            imwrite(I4,PrintNameList(q,i).name,'tif');
        elseif ImageChosen == 4
            imwrite(I5,PrintNameList(q,i).name,'tif');
        elseif ImageChosen == 5
            imwrite(I6,PrintNameList(q,i).name,'tif');
        elseif ImageChosen == 6
            imwrite(I7,PrintNameList(q,i).name,'tif');
        elseif ImageChosen == 7
            imwrite(A1,PrintNameList(q,i).name,'tif');
        elseif ImageChosen == 8
            imwrite(I8,PrintNameList(q,i).name,'tif');
        elseif ImageChosen == 9
            imwrite(I9,PrintNameList(q,i).name,'tif');
        elseif ImageChosen == 10
            imwrite(I10,PrintNameList(q,i).name,'tif');
        elseif ImageChosen == 11
            imwrite(I11,PrintNameList(q,i).name,'tif');
        elseif ImageChosen == 12
            imwrite(I12,PrintNameList(q,i).name,'tif');
        elseif ImageChosen == 13
            imwrite(I13,PrintNameList(q,i).name,'tif');
        end
    end
    PrintList = size(Index,1);
else
    PrintList = 0;
end
```

GeneratingPrintName:

```
function [PrintNameList] = GeneratingPrintName(PrintIndex,s)

%%%%%%%%%%%%%%%%%%%%%%%%%%%%%%%%%%%%%%%%%%%%%%%%%%%%%%%%%%%%%%
%%%%%%%%%%%%%%%%%%%%%%%%%%
%This function receive an Index, which show the chosen matrices, and the
%number of images being processed.
%This function then produce the list of names of the chosen matrices
%PrintIndex:    The index of chosen matrices (m x 1)
%s:             The number of images being processed (1 x 1)
%%%%%%%%%%%%%%%%%%%%%%%%%%%%%%%%%%%%%%%%%%%%%%%%%%%%%%%%%%%%%%
%%%%%%%%%%%%%%%%%%%%%%%%%%

Index = find(PrintIndex>0);
Cond = isempty(Index);

if Cond == 0
    for i = 1:size(Index,1)
        ImageChosen = Index(i,1);
        if ImageChosen == 1
            PName = 'I2';
        elseif ImageChosen == 2
            PName = 'I3';
        elseif ImageChosen == 3
            PName = 'I4';
        elseif ImageChosen == 4
            PName = 'I5';
        elseif ImageChosen == 5
            PName = 'I6';
        elseif ImageChosen == 6
            PName = 'I7';
        elseif ImageChosen == 7
            PName = 'A1';
```

```
        elseif ImageChosen == 8
            PName = 'I8';
        elseif ImageChosen == 9
            PName = 'I9';
        elseif ImageChosen == 10
            PName = 'I10';
        elseif ImageChosen == 11
            PName = 'I11';
        elseif ImageChosen == 12
            PName = 'I12';
        elseif ImageChosen == 13
            PName = 'I13';
        end
        for j = 1:s
            PrintName = sprintf('%s-%03d.tif',PName,j);
            PrintNameList(j,i).name = PrintName;
        end
    end
else
    PrintNameList = 'NO PRINT CHOSEN';

end
```

Main:

```
clear all;
clc

%% ==================== List of image matrices ============================
%I  = original image
%I2 = original image smoothed by mean filter (6x)
%I3 = image of pixels that above the threshold (T)
%I4 = image of pixels that above the threshold with the holes filled
%I5 = image of the thresholded nucleus in black background plus intensity
%     redistributed
%I6 = downsampled I5 into 128x128 (by a factor of 4)
%I7 = SOBEL image (logical)
%A1 = SOBEL image (uint8)
%I8 = image of pixels that above the threshold from 128x128 image (I6)
%I9 = image of pixels that above the threshold from 128x128 image with the
%     holes filled
%I10 = the inner part of the nucleus (Region of Interest or ROI)
%I11 = perimeter of the ROI
%I12 = image of the SOBEL edge within the ROI
%I13 = image of the SOBEL edge within the ROI (I12) plus the perimeter of
%      previous ROI (I11)

%% ================== INPUT ==============================================
%These are the required inputs for the algorithm.
%XLfilename:    The name given to the EXCEL file to be produced, this will
%               contain the area, edge count and edge density from each
%               image.
%filenames:     This will search the folder for the images name specified.
%
%If you want to print out an image matrix, give the value 1. If not
%required, give the value 0.
%
%PixRedFaxtor:  Image reduction factor.
%SobelThresh:   The threshold value for the SOBEL edge detection.
%=========================================================================

XLfilename = 'Results AREA EDGECOUNT EDGEDENSITY.xls';
filenames = dir('*.tif');

PrintI2 = 0;
PrintI3 = 0;
PrintI4 = 0;
PrintI5 = 0;
```

```
PrintI6 = 0;
PrintI7 = 0;
PrintA1 = 0;
PrintI8 = 0;
PrintI9 = 0;
PrintI10 = 0;
PrintI11 = 0;
PrintI12 = 0;
PrintI13 = 0;

PixRedFactor = 8;
SobelThresh = 0.09;

%% ===== Producing file names for image matrices to be printed out =======

PrintIndex = [PrintI2;PrintI3;PrintI4;PrintI5;PrintI6;PrintI7;PrintA1;...
    PrintI8;PrintI9;PrintI10;PrintI11;PrintI12;PrintI13];
s = numel(filenames);
[PrintNameList] = GeneratingPrintName(PrintIndex,s);

%% =================== CORE algorithm ======================================

Arealist = zeros(s,1);
edgecountlist = zeros(s,1);
edgedenlist = zeros(s,1);

for q=1:numel(filenames)
    %Load image
    I = imread(filenames(q).name);

    %Acquire threshold value for I
    [T] = ThreshMode(I);

    %Image average smoothening by (i)th times
    I2 = I;
    for i = 1:6
        h = fspecial('average');
        I2 = imfilter(I2,h);
    end

    %Thereshold application to I2
    [I3] = ApplyThresh(I2,T);
    I3 = logical(I3);

    %Hole-filling algorithm
    I4 = imfill(I3,'holes');

    %Extract the nucleus from the original image to a black background
    [I5] = ExtractImage(I,I4);
    I5 = uint8(I5);

    %Intensity redistribution for I5
    A = max(max(I5));
    B = double(I5);
    C = double(A);
    I5 = (B/C)*255;
    I5 = uint8(I5);

    %Image reduction by a factor of 4 (1/4 = 0.25)
    PixRed = 1/PixRedFactor;
    I6 = imresize(I5,PixRed);

    %Intensity redistribution for I6
    A = max(max(I6));
    B = double(I6);
    C = double(A);
    I6 = (B/C)*255;
    I6 = uint8(I6);
```

```
    %SOBEL edge detection application
    I7 = edge(I6,'sobel',SobelThresh);
    A1 = uint8(I7);
    A1 = A1*255;

    %Acquire threshold value for I6
    clear T
    [T] = ThreshMode(I6);

    %Threshold application to I6
    [I8] = ApplyThresh(I6,T);
    I8 = logical(I8);

    %Hole-filling algorithm
    I9 = imfill(I8,'holes');

    %Perimeter subtraction by (n)th times
    I10 = I9;
    n = 2;
    for i = 1:n
        I11 = bwperim(I10);
        I10 = I10-I11;
        I10 = logical(I10);
    end

    %Extract the SOBEL edge inside the nucleus into a black background
    [I12] = ExtractImage(I7,I10);
    I12 = logical(I12);
    I13 = I12+I11;
    I13 = uint8(I13);
    I13 = I13*255;

    %Nucleus area
    [row,column,int] = find(I10>0);
    S = length(row);
    Area = S;
    Arealist(q,1) = Area;

    %Edge count
    edgecount = sum(sum(I12));
    edgecountlist(q,1) = edgecount;

    %Edge density (i.e. chromatin condensation parameter)
    edgeden = (edgecount/Area)*100;
    edgedenlist(q,1) = edgeden;

    I12 = uint8(I12);
    I12 = I12*255;
    [PrintList] = GeneratingPrint(PrintIndex,PrintNameList,I2,I3,I4,...
        I5,I6,I7,A1,I8,I9,I10,I11,I12,I13);
end

xlswrite(XLfilename,Arealist,1);
xlswrite(XLfilename,edgecountlist,2);
xlswrite(XLfilename,edgedenlist,3);
```

ThreshMode:

```
function [Thresh] = ThreshMode(I)

%%%%%%%%%%%%%%%%%%%%%%%%%%%%%%%%%%%%%%%%%%%%%%%%%%%%%%%%%%%%%%%%%%%%%%%%%%%%%%%%%%%%
%%%%%%%%%%%%%%%%%%%%%%%%%%%%
%This function receive the image to be thresholded.
%This function then produce a threshold value by the mode method.
%I: image to be thresholded
```

```
%%%%%%%%%%%%%%%%%%%%%%%%%%%%%%%%%%%%%%%%%%%%%%%%%%%%%%%%%%%%%%%%
%%%%%%%%%%%%%

I = double(I);
MaxInt = max(max(I));
MaxInt = single(MaxInt);

H = hist(I(:),0:MaxInt);

Iteration = 0;
Cond = 0;
Thresh = 0;

while Cond == 0
    F = ones(1,3)/3;
    H = conv2(H,F,'same');

    Sy = size(H,2);
    Peak = 0;

    for i = 2:Sy-1
        if Peak < 3
            if H(i-1)<H(i) && H(i+1)<H(i)
                Peak = Peak+1;
            end
        end
    end

    if Peak > 2
        Cond = 0;
    else
        Cond = 1;
    end

    Iteration = Iteration + 1;

    if Iteration > 10000
        Thresh = 0;
        return
    end
end

for j = 2:MaxInt
  if H(j-1)>H(j) && H(j+1)>H(j)
    Thresh = j-1;
  end
end
```
